# Steatohepatitis-induced vascular niche alterations promote melanoma metastasis

**DOI:** 10.1186/s40170-025-00374-6

**Published:** 2025-01-28

**Authors:** Johannes Hoffmann, Julia Schüler, Bianca Dietsch, Sina Wietje Kürschner-Zacharias, Carsten Sticht, Felix A. Trogisch, Maren Schreitmüller, Tinja Baljkas, Kai Schledzewski, Manuel Reinhart, Sebastian A. Wohlfeil, Manuel Winkler, Christian David Schmid, Joerg Heineke, Cyrill Géraud, Sergij Goerdt, Philipp-Sebastian Reiners-Koch, Victor Olsavszky

**Affiliations:** 1 Department of Dermatology, Venereology and Allergology, University Medical Center and Medical Faculty Mannheim, Heidelberg University, Theodor-Kutzer-Ufer 1-3, Mannheim, 68167 Germany; 2 European Center for Angioscience (ECAS), Medical Faculty Mannheim, Heidelberg University, Mannheim, Germany; 3 Section of Clinical and Molecular Dermatology, Department of Dermatology, Venereology and Allergy, Medical Faculty Mannheim, University Medical Centerand, Heidelberg University , Mannheim, Germany; 4 Core Facility Platform Mannheim (CFPM), Medical Faculty Mannheim, NGS Core Facility, Heidelberg University, Mannheim, Germany; 5 Core Facility Platform Mannheim (CFPM), Cardiac Imaging Center, Mannheim, Faculty of Medicine, Heidelberg University, Mannheim, 68167 Germany; 6 DZHK (German Center for Cardiovascular Research), Partner Site Heidelberg/Mannheim, Mannheim, Germany; 7 Skin Cancer Unit, German Cancer Research Center (DKFZ), Heidelberg, 69120 Germany

**Keywords:** Cutaneous malignant melanoma, Hepatic metastasis, Metabolic dysfunction-associated steatohepatitis, Early vascular alterations, Liver sinusoidal endothelial cells

## Abstract

**Background:**

In malignant melanoma, liver metastases significantly reduce survival, even despite highly effective new therapies. Given the increase in metabolic liver diseases such as metabolic dysfunction-associated steatotic liver disease (MASLD) and metabolic dysfunction-associated steatohepatitis (MASH), this study investigated the impact of liver sinusoidal endothelial cell (LSEC)-specific alterations in MASLD/MASH on hepatic melanoma metastasis.

**Methods:**

Mice were fed a choline-deficient L-amino acid-defined (CDAA) diet for ten weeks to induce MASH-associated liver fibrosis, or a CDAA diet or a high fat diet (HFD) for shorter periods of time to induce early steatosis-associated alterations. Liver metastasis formation was assessed using melanoma cell lines B16F10*Luc2* and Wt31. LSEC-specific GATA4 knockout mice (*Gata4*^LSEC−KO/BL^) developing MASH-like liver fibrosis without steatosis via a pathogenic angiocrine switch were included to compare the impact of liver fibrosis versus hepatic steatosis on hepatic melanoma metastasis. Bulk RNA-Seq of isolated LSECs from CDAA-fed and control mice was performed. Levels of adhesion molecules (VCAM1, ICAM1, E-selectin) were monitored, and ICAM1 and VCAM1 antibody therapy was employed.

**Results:**

Feeding a CDAA diet, in contrast to a HFD, led to increased metastasis before the development of liver fibrosis. *Gata4*^LSEC−KO/BL^ mice characterized by vascular changes ensuing perisinusoidal liver fibrosis without steatosis also exhibited increased metastasis. Early molecular alterations in the hepatic vascular niche, rather than fibrosis or steatosis, correlated with metastasis, as shown by LSEC dedifferentiation and upregulation of endothelial adhesion molecules. The metastatic process in CDAA-fed mice was also dependent on the respective melanoma cell lines used and on the route of their metastatic spread. ICAM1 inhibition, but not VCAM1 inhibition reduced melanoma cell retention.

**Conclusion:**

We discovered that the hepatic vascular niche acts as a delicate sensor to even short-term nutritional alterations during the development of MASLD/MASH. The dynamic adaptations to the metabolic challenges of developing MASLD/MASH caused an early shift from the normal hepatic vascular niche to a pre-metastatic vascular niche that promoted hepatic melanoma metastasis in the context of cell-autonomous and acquired melanoma cell features. Altogether, our findings provide a potential avenue for angiotargeted therapies to prevent hepatic melanoma metastasis.

**Supplementary Information:**

The online version contains supplementary material available at 10.1186/s40170-025-00374-6.

## Background

Cutaneous malignant melanoma (MM) is a highly aggressive cancer of melanocytic origin with high metastatic rates accountable for over 90% of skin cancer mortality [1]. Common sites of MM metastasis include skin, lungs, brain, bones, and digestive tract. Brain, liver, and lung metastases are associated with the highest mortality [2]. Clinically, 18%–36% of MM metastases are found in the lungs, and 14%–20% in the liver [3]. Autopsy series revealed liver metastases even in up to 58% of melanoma patients [4]. Liver metastases in MM are associated with a poor prognosis and with resistance to treatment with immune checkpoint inhibitors or targeted therapies [5, 6]. Therefore, liver metastases pose a major challenge for improving overall survival in metastatic MM patients [5, 7].

Over a century ago, Stephen Paget first proposed the "seed and soil" hypothesis suggesting that certain tumors preferentially metastasize to specific organs [8]. The underlying metastatic cycle [9] involves repeated interactions between the metastatic niche of the target organ and the tumor cells, with tumor-derived systemic signals converting organ-specific niches into pre-metastatic niches ready to support metastasis.

Among various organs, the liver displays a unique vascular niche with capillary vessels lined by highly specialized liver sinusoidal endothelial cells (LSECs). LSECs, can facilitate hepatic metastasis and actively participate in development of the preformed organ-specific hepatic vascular niche into a pre-metastatic and finally a metastatic vascular niche. The mechanisms that drive the pro-metastatic function of the hepatic vascular niche comprise expression of adhesion molecules [10] and secretion of angiocrine factors [11] by LSECs. Our group has previously shown that the unique molecular repertoire of LSECs and the secretion of specialized angiocrine factors control normal organ development and organ homeostasis in the liver [12, 13]; vice versa, dedifferentiation of LSECs was found to cause liver pathologies [13, 14]. Therefore, we hypothesized that local pathological changes may activate the hepatic vascular niche and unintentionally promote MM metastasis to the liver.

Metabolic dysfunction-associated steatotic liver disease (MASLD) and metabolic dysfunction-associated steatohepatitis (MASH), which have become the most common chronic liver diseases in the western world, may be such a local factor promoting liver metastasis [15]. Although direct clinical and epidemiological confirmation is lacking, circumstantial evidence demonstrates that obesity, BMI and MASLD/MASH may influence melanoma metastasis to the liver [16]. Of note, in malignant melanoma, there is a well-established association with overweight and obesity, particularly in men [17]. In addition, a body mass index (BMI) > 35 is an independent risk factor for melanoma metastasis [18]. Obesity-related factors such as systemic inflammation and metabolic syndrome have been suggested to play a role in melanoma progression [19, 20], further complicating the understanding of metastatic dynamics in the context of MASLD/MASH.

Hitherto, however, the alterations that constitute the pre-metastatic vascular niche induced in the liver by MASLD/MASH have not been comprehensively analyzed. As LSECs have been shown to play an important role as gatekeepers in progression from MASLD to early MASH [21] and as LSECs are important players in the development of perisinusoidal liver fibrosis [14], we here analyzed melanoma metastasis during the different steps in progression of MASLD to advanced MASH using diet-induced and genetic models of MASH and MASH-associated liver fibrosis.

## Methods

### Animals

C57Bl/6NRj (RRID: MGI:6,236,253) mice were purchased from Janvier Labs (Le Genest-Saint-Isle, France) and housed under specific pathogen-free conditions in individually ventilated plastic cages (Sealsafe plus DGMTM, Techniplast, Italy) with wood litter (Ssniff, Soest, Germany, H0234-20), adjusted air temperature (21 °C) and 50% relative humidity and acclimatized for a week. All mice were housed in a 12h/12h day/night cycle with free access to water and were fed ad libitum with a standard rodent diet (Ssniff, Soest, Germany, V1534-000). Mice that were included in the CDAA- or HFD-feeding cohorts were 10 weeks of age and were fed ad libitum with CDAA diet (Ssniff, Soest, Germany, E15666-94) or HFD diet (Ssniff, Soest, Germany, E15742-34) with free access to water.

### Mouse tumor model

For B16F10*Luc2* and Wt31 liver metastasis experiments, mice were anesthetized and a laparotomy was performed. 1.5 × 10^5^ B16F10*Luc2* cells or 1.5 × 10^4^ Wt31 cells suspended in 60 µl 1X DPBS were injected intrasplenically. 15 min after injection a splenectomy was performed. The peritoneum and skin were closed with suture (Vicryl 6.0, Ethicon) and for rehydration, all operated mice received prewarmed (37 °C) sterile 0.9% NaCl solution subcutaneously. For analgetic treatment, mice were given carprofen or buprenorphine and were monitored frequently. Mice were sacrificed 14 days after B16F10*Luc2* or 19 days after Wt31 cell injection. Organs were removed and analyzed for melanoma colonization. For generation of Wt31 liver metastases via intravenous injection, mice were warmed up in a warming chamber at 37 °C for 10 min. 1.25 × 10^6^ Wt31 cells suspended in 100 µL 1X DPBS were injected into the tail vein. The animals were sacrificed and analyzed 19 days after cell injection. Mice with abdominal metastases were excluded from the analysis. If the termination criteria in the animal welfare application were met, the animals were not included in the analysis. Investigators were not blinded during the experiments and analysis of results.

### Tumor cell retention assay

Retention tests for the B16F10*Luc2* cell line were carried out by our group as published [23]. For retention tests with Dil-labeled Wt31 cells (Vybrant™ Dil Cell-Labeling Solutions (V22885)), 8 × 10^4^ Dil-labeled Wt31 cells suspended in 60 µL 1X DPBS were injected intrasplenically. After injection, the spleen was left in the mouse, and all further steps were performed as previously described in the methodological section 'Mouse tumor model'. The fluorescence signal in the liver was measured ex vivo after 90 min. For the intravenous retention assay, 4 × 10^4^ Dil-labeled Wt31 cells suspended in 100 µL of 1X DPBS were injected intravenously via the tail vein and the fluorescence signal in the liver was measured ex vivo after 90 min. For antibody therapy, the mice were injected intraperitoneally with 500 µg anti-ICAM1 antibody (RRID: AB_1107661) [22], 200 µg anti-VCAM1 antibody (RRID: AB_1107572) or an InVivoMAb rat IgG2b isotype control 24 h prior to tumour cell injection. The investigators were not blinded during the experiments and analysis of results.

### Animal ethics and study approval

All animals received humane care in compliance with the Guide for the Care and Use of Laboratory Animals published by the National Academy of Sciences and the animal ethics committee of Baden Wuerttemberg (Regierungspraesidium Karlsruhe) approved all animal experiments.

### Cell lines

All cancer cell lines used for metastasis experiments were of murine origin and are well established in our laboratory for metastasis experiments as previously mentioned [23]. The B16F10*Luc2* cell line was purchased from Perkin Elmer (PerkinElmer, MA, United States, RRID: CVCL_A4CJ, passage number 10–12). O. Sansom donated the transformed mouse melanoma cell line Wt31 (Beatson Institute for Cancer Research, Glasgow, United Kingdom, passage number 7–9). All cell lines were regularly tested mycoplasma-free by PCR. For this purpose, the PCR Mycoplasma Detection Kit (Biozol, ABM-G238) was used according to the manufacturer's instructions. STR sequencing (Microsynth AG, Balgach, Switzerland) was performed for cell authentication and confirmed unique profiles of all cell lines used. Cells were also distinguished by pigmentation status, morphology or bioluminescence.

### Cell size determination

Cancer cell lines were cultured as described in 'Cell lines' above and suspended in 1X Dulbecco's phosphate buffered saline (DPBS) supplemented with 5% (v/v) Gibco™ FBS for measurements. For determination of the relative cell size, a BD Canto II flow cytometer (RRID: SCR_018056) coupled with FACS Diva software (BD Biosciences, NJ, USA) was used. The gating strategy included the elimination of duplicates followed by gating on living cells using SYTOX™ Red Dead Cell Stain (Thermo Fisher Scientific, MA, United States, S34859). The median of the FSC-W was used as a parameter to determine relative cell size. All values were normalized to the lowest value for B16F10*Luc2*.

### Quantification of metastatic burden

The number of metastases on the liver surface was counted visually and by hand for all cell lines used. The livers were divided into individual lobes, the metastases were counted individually, and the total number was calculated. An image of the top and bottom of each liver was used to measure the metastatic area. The metastatic area was determined using Threshold in ImageJ (RRID: SCR_003070) and divided by the total liver area, also determined using Threshold in ImageJ. The result was calculated as the percentage of metastatic area in relation to the total liver area.

### Ex vivo Bioluminescence imaging (BLI)

Live mice were injected intraperitoneally with 150 µl D-Luciferin (potassium salt, 30 mg/ml) and imaged after 10 min anesthetized with isoflurane using an IVIS Lumina III In Vivo Imaging System (PerkinElmer, MA, United States, RRID:SCR_025239), exposure time 45 s, binning medium, field of view 12.5, f/stop 1, open filter. To quantify the BLI signal, Living Image 4.7.3 ® (PerkinElmer, MA, United States, RRID: SCR_014247) was used with regions of interest around the livers. The total flux (p/sec) signal was measured and displayed as radiance (p/sec/cm^2^/sr). The anterior and posterior sides of the livers were measured, and the mean value was calculated and used for the statistical analysis.

### Genetic mouse model

For the generation of LSEC-specific conditional knockout mice *Clec4g*-icre driver mice (C57BL/6N-Tg(*Clec4g-icre*)1.1Sgoe, MGI:6,280,453) were crossed to *Gata4* floxed mice (B6.129 *Gata4*^tm1.1Sad/J^), bearing a loxP site downstream of exon 3 and a loxP site upstream of exon 5. Mice bearing the genotype *Clec4g-icre*^tg/0^ × *Gata4*^fl/fl^ indicating homozygous recombination were denoted as *Gata4*^LSEC−KO/BL^. Littermates bearing the genotype *Clec4g-icre*^0/0^ × *Gata4*^fl/fl^ were used as controls. All *Gata4* control and mutant mice analyzed were 3-month-old females.

### RNA in situ hybridization

In situ hybridization (ISH) was performed on FFPE tissue Sects. (3 μm) according to the manufacturer’s protocols. The RNAscope 2.5 HD Red kit (322,350, Advanced Cell Diagnostics, Newark, CA, USA) was used with mouse specific probes against the mouse *Pdgfb* gene (424,651, Advanced Cell Diagnostics) and the mouse *Ppib* (*Cyclophilin B*) gene as positive control.

### RNA fluorescence in situ hybridization

RNA fluorescence in situ hybridization (FISH) was performed on FFPE tissue Sects. (3 μm) according to the manufacturer’s protocols. The RNAscope 2.5 HD Duplex kit (Advanced Cell Diagnostics, 322,430) was used with mouse specific probes against the positive control mouse *Ppib* (*Cyclophilin B*) gene, mouse *Cdh5*-Channel 2 (312,531-C2—NM_009868.4), mouse *Gpnmb*-Channel 1 (489,511—NM_053110.4), mouse *Sele*-Channel 1 (438,621—NM_011345.2).

### Immunohistochemistry and immunofluorescence staining

Deparaffinization and rehydration of paraffin Sects. (3 μm) were performed according to standard protocols. Antigen retrieval was performed with epitope retrieval solution (Zytomed Systems, Germany) at pH 6, pH 8 or pH 9.

Cryo Sects. (8 μm) were fixed with 4% PFA for 10 min after cutting the fresh frozen tissue and air drying. A blocking step was performed for 45 min with 5% normal donkey serum (Dianova, Hamburg, Germany, 017–000–121).

For immunofluorescence staining, the primary antibody was diluted in Dako antibody diluent (Agilent Technologies, S202230-2) an incubated overnight at 4 °C in a humidity chamber. The secondary antibody was diluted in Dako antibody diluent (Agilent Technologies, S202230-2) and applied after washing three times with PBS for 1 h at room temperature. Stained sections were covered with fluorescent Dako mounting medium (Dako, Agilent Technologies, USA) and a cover slip. For staining with hematoxylin and eosin (H&E) as well as Picrosirius Red staining, formalin-fixed, paraffin-embedded specimens were processed according to the standard protocols of the manufacturer. For staining with Oil red O, fresh frozen cryo sections were processed according to the standard protocols of the manufacturer and counter stained with hematoxylin solution.

For immunohistochemical staining, FFPE sections were deparaffinised and rehydrated as described above. The sections were then blocked with Dako Real Peroxidase Blocking Solution (Agilent Technologies, S2023) for 10 min and the primary antibody was diluted in Dako Antibody Diluent (Agilent Technologies, S202230-2) and incubated overnight at 4 °C in a humidity chamber. After three washes with PBS, sections were incubated with Dako EnVision + System, HRP-conjugated anti-rabbit polymer (Agilent Technologies, RRID: AB_2630375) for 1 h at room temperature. After three washes with PBS, the sections were incubated with Dako Liquid DAB + Substrate (Agilent Technologies, K3468) for 8 min, counterstained with haematoxylin solution, Gill No. 1 (Sigma-Aldrich, GHS132-1L) for 4 min and mounted with Dako aqueous mounting medium (Agilent Technologies, S3025).

### Scanning electron microscopy

Roller pump-mediated perfusion fixation through the portal vein of total liver was performed in situ with 4% formaldehyde (Carl Roth, Germany, P087) for pre-perfusion followed by 1.5% glutaraldehyde (Electron Microscopy Sciences, United States, 16,360) in cacodylate buffer (Electron Microscopy Sciences, United States, 11,653) for 5 min. After the liver has been removed, it was washed in cacodylate buffer and cut into 1 mm × 1 mm × 5 mm strips. The tissue blocks were transferred in 1% osmium tetroxide (Electron Microscopy Sciences, United States, 19,110) in cacodylate buffer and incubated for 1 h on ice in the dark. Tissue blocks were washed twice with cacodylate buffer. For sample dehydration, the tissue blocks were placed in 30% acetone, 50% acetone, 70% acetone, 90% acetone and twice in 100% acetone (Honeywell, 32,201-1L) for 10 min each. The Leica EM CPD 300 AUTO Critical Point Dryer (Leica Microsystems, Germany) was used for critical point drying. Tissue blocks were gently mounted on 0.5'' SEM Pin Stubs (Agar Scientific Ltd, UK, AGG301F) and sputter coated with a 10 nm thick layer of gold/palladium in a Leica EM ACE600 Carbon Coater (Leica Microsystems, Germany, RRID: SCR_020237). Microscopy was performed using a Zeiss Leo 1530 scanning electron microscope (Carl Zeiss AG, Germany) coupled to Zeiss SmartSEM software (Carl Zeiss AG, Germany).

### Echocardiography and doppler analyses

Liver echocardiography was performed 1 or 7 days after the start of the dietary regimen using a high-frequency ultrasonography device (VEVO 3100, VisualSonics, RRID: SCR_022152). Before investigation, mice were anesthetized with 3% isoflurane (CP-Pharma) in 1 L/min oxygen and maintained at 1% isoflurane. The portal vein was identified in transverse abdominal view and recorded in elongated parasternal long axis. The vessel branching off to the right at the level of the right hepatic lobe, also marked as the "1st generation branch vessel", was identified as the right median portal vein and recorded in the long axis. Flow analyses were conducted utilizing Color and Pulsed-wave Doppler imaging. Diameters were measured in M- or B-mode, respectively.

### Isolation of liver sinusoidal endothelial cells

For isolation of LSECs murine livers were perfused via the portal vein with 0.05% Collagenase in Ca^2+^-deprived medium followed by mincing using scissors. After collagenase digestion of the liver in a 37 °C water bath, non-parenchymal cells (NPCs) were separated from hepatocytes by low-speed centrifugation. The NPC cell suspension was filtered through a 250 µm mesh and then passed through a 100 µm cell strainer to obtain a single cell suspension. A 19.3% Nycodenz gradient followed by Magnetic-activated cell sorting (MACS) using anti-LSEC (CD146^+^) MicroBeads from Miltenyi Biotec was used for positive selection of LSEC according to the manufacturer’s instruction. For bulk RNA-Seq, MACS-purified LSEC did not undergo further purification by FACS sorting. The purity of the isolated LSECs was analysed by using a BD Canto II flow cytometer (RRID: SCR_018056) after the MACS-bead sorting step. For staining, Live/Dead stain (565,388, RRID: AB_2869673, BD Horizon) was used for alive cells, CD31 PerPC/Cyanine5.5 antibody (102,522, RRID: AB_2566761, clone MEC13.3, BioLegend) and LYVE-1 PE antibody (FAB2125P, RRID: AB_10889020, clone 223,322, R&D Systems) for LSECs, and CD11b BV510 antibody (101,245, RRID: AB_2561390, Clone M1/70, BioLegend) for myeloid cells. The proportions of LYVE1^+^/CD31^+^ and CD11b^+^ cells were assessed separately for chow-fed and CDAA-fed mice to evaluate potential contamination with myeloid cells. Data were analyzed using FlowJo v. 10.7.2 (Becton Dickinson, RRID: SCR 008520).

### Organ dissection, cryopreservation and paraffin embedding

The mice were sacrificed by cervical dislocation and the body and organ weights were measured. Liver tissue samples were harvested, washed in 1X DPBS and photographed using a Nikon D5600 with a scale. Organs were then either embedded in Tissue-Tek® O.C.T.™ compound (Sakura Finetek) and frozen in liquid nitrogen or fixed in 4% formaldehyde, phosphate buffered, pH 7 at room temperature, followed by paraffin embedding according to routine protocols.

### Antibodies

Primary antibodies: Goat anti-PODXL (AF1556, R&D Systems, USA, RRID: AB_354858), rabbit anti-Stabilin-2 peptide 15 antibody [24], goat anti-LYVE1 (AF2125, R&D Systems, USA, RRID: AB_2297188), rabbit anti-LYVE1 (ab36993, Abcam, GB, RRID: AB_2138663), rat anti-Endomucin (14–5851-82, Thermo Fisher Scientific, USA, RRID: AB_891527), rabbit anti-ICAM1 (10,020–1-AP, Proteintech, USA, RRID: AB_2121773), goat anti-VCAM1/CD106 (AF643, R&D Systems, USA, RRID: AB_355499), rat anti-CLEC4F/CLECSF13 (MAB2784, R&D Systems, USA, RRID: AB_2081338), rat anti-Sialoadhesin/CD169 (ab53443, Abcam, GB, RRID: AB_2189028), rabbit anti-TRP-2 (ab74073, Abcam, GB, RRID: AB_1524517), rat anti-CD 31 (DIA-310, Dianova, Germany, RRID: AB_2631039), guinea pig anti-Glutamine Synthetase (367,005, Synaptic System, Germany, RRID: AB_2620128).

Secondary antibodies: Alexa-Fluor 488, Alexa-Fluor 647 and Cy3-conjugated secondary antibodies were purchased from Dianova (Hamburg, Germany).

### Image processing

Images of routine histological examinations and immunofluorescence staining were acquired with an Eclipse Ni-E motorized upright microscope (Nikon Instruments Europe BV, Amsterdam, Netherlands) using 10x, 20x or 40x CFI Plan Apochromat Lambda objectives, a Sola Lumencore light engine, a DS-Ri2 high-definition color camera and a DS-Qi2 high-definition monochrome camera. The system was controlled with NIS-Elements AR 5.30.6 software (Nikon Instruments, Tokyo, Japan).

The immunohistochemical staining of TRP-2 was scanned using an Axio Scan.Z1 automatic slide scanner (Zeiss, Jena, Germany, RRID: SCR_020927) operating under bright field conditions, 20% light source intensity, with a 10x/0.45 plan apochromat and a Hitachi HV-F202SCL camera. Images were exported with Zen Software (Zeiss, Jena, Germany) and analyzed with ImageJ. The metastatic area was determined using Threshold in ImageJ and divided by the total liver area, also determined using threshold in ImageJ. The result was calculated as the mean metastatic area.

Three representative areas per sample underwent quantification for the immunofluorescence or immunohistochemistry images. The background of the IF colorations in all images was reduced with the rolling ball background subtraction tool of the NIS-Elements Advanced Research (AR) software before becoming focused. The quantification of the images was carried out using the ImageJ software with a pre-programmed threshold suitable for the analysis. The percentage area and mean fluorescence intensity were analyzed using GraphPad Prism, applying statistical methods.

The H&E and Picrosirius Red staining brightfield images underwent processing with ImageJ tool. Quantification of the Oil Red O images followed color deconvolution and thresholding techniques for area determination.

### Blood parameters

Blood samples were taken from retrobulbar venous plexus during day cycle in lithium heparin tubes (Microvette 500 LH, 20.1345.100, Sarstedt, Nümbrecht, Germany). Plasma was separated (centrifugation at 2000 × g^−1^ for 5 min) and analyzed for ALT, AST, cholesterol, cholinesterase, and triglyceride (Roche cobas c 311 analyzer, RRID: SCR_024559, Roche Diagnostics, Basel, Switzerland) according to the manufacturer’s recommendations.

### Triglyceride assay

Snap frozen liver tissue (approximately 100 mg) was homogenized in 5% NP-40 solution (74,385, Merck, Darmstadt, Germany) and heated for 5 min in a shaking dry incubator (ThermoMixer C, Eppendorf, Hamburg, Germany) at 80–100 °C. After cooling to room temperature, heating was repeated to solubilize all triglycerides. After centrifugation for 2 min at top speed (Centrifuge 5417 R, RRID: SCR_019847, Eppendorf) the supernatant was diluted tenfold in distilled water and used to determine the triglyceride content based on the protocol of the Triglyceride Quantification Colorimetric/Fluorometric Kit manufacturer (BioVision, Mountain View, CA, United States, K622).

### RNA isolation and sequencing

After LSEC isolation, the RNA was extracted using the innuPREP RNA Mini Kit (Analytik Jena, Jena, Germany, 845-KS-2080250). For whole liver RNA, a piece of liver was homogenised with 450 µl Lysis solution RL (provided by innuPREP RNA Mini Kit) using Precellys homogenizer (5000 rpm, 20 s) with Precellys Lysing Kit CKMix (2 ml tube with ceramic beads). The RNA concentration and quality were then measured using a NanoPhotometer NP80 (Implen, Munich, Germany) and a 2100 Bioanalyzer (Agilent Technologies, Santa Clara, USA, RRID: SCR_019715). The library preparation and the sequencing were performed by BGI (Hong Kong, China).

### RNA data analysis

RNA-seq data processing was performed with R (version 3.6.3) and bioconductor (version 3.9, RRID: SCR_006442) in Rstudio (version 1.1.463, RRID:SCR_000432). Quality control of clean sequencing reads was performed using FastQC (Babraham Bioinformatics, RRID: SCR_014583). Low-quality reads were removed using trim_galore (version 0.6.4, RRID: SCR_011847). The resulting reads were aligned to the mouse genome version GRCm38.p6 and counted using kallisto version 0.46.1 (RRID: SCR_016582). The count data was transformed to log2-counts per million (logCPM) using the voom-function from the limma package. Differential expression analysis was performed using the limma package in R. A false positive rate of α = 0.05 with FDR correction was taken as the level of significance. Volcano plots were created using GraphPad Prism (version 8.4.3, RRID: SCR_002798) and heatmaps were created using ggplot2 package (version 2.2.1, RRID: SCR_014601) and the complexHeatmap package (version 2.0.0, RRID: SCR_017270). For enrichment analysis, we used the fgsea (RRID: SCR_020938), the enrichmentbrowser, and the Enrichr (RRID: SCR_001575) packages.

The pathway analysis was made with fgsea package and the enrichment browser package in R using the pathway information from KEGG database (https://www.genome.jp/kegg/pathway.html, RRID: SCR_018145).

### Statistic

Statistical significance was tested using the GraphPad Prism software (version 8.4.3, RRID: SCR_002798). The Shapiro–Wilk test was used to assess the normal distribution and the F-test was used to assess the equality of variances with equal variance of the data. Assuming a Gaussian distribution, the unpaired two-tailed Student's t-test or Welch's t-test was used to statistically analyze two groups. For non-normally distributed data, the Mann–Whitney U test was used. For the statistical comparison of more than two groups with one independent variable, the one-way ANOVA (Bonferroni post-hoc test) was used, while for the statistical comparison of more than two groups with two independent variables, the two-way ANOVA (Tukey post-hoc test) was used. Data are presented as mean values with standard deviation (SD). Asterisks indicate the significance level: * *p* ≤ 0.05, ** *p* < 0.01, *** *p* < 0.001, **** *p* < 0.0001. Mice were randomly selected for the different feeding regimes, and quantifications of the in vivo experiments were performed with at least five biological replicates. The PROC POWER procedure in the SAS 9.4 statistics program with the TWOSAMPLEMEANS TEST specification was used to calculate the sample size. This was based on a two-sided test with a significance level of α = 0.05 and a statistical POWER of 80%. For quantitative differences of 40% for two groups and a standard deviation of 25%, the calculation program used resulted in.

two sample means test = diff.

mean diff = 40.

stddev = 25.

npergroup = .

power = 0.80;

a sample size of *n* = 8.

## Results

### Diet-induced fibrotic steatohepatitis promotes melanoma metastasis

**Fig. 1:**
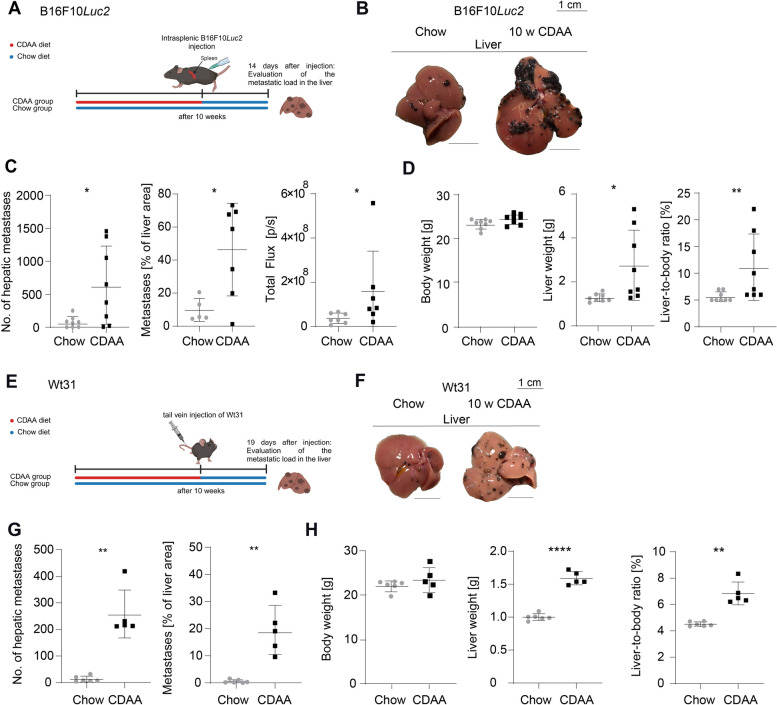
Enhanced B16F10*Luc2* and Wt31 melanoma metastasis formation after 10 weeks of CDAA diet. **A** Setup for metastasis formation in CDAA diet-induced MASH model after intrasplenic injection of B16F10*Luc2* cells and assessment of metastatic burden 14 days later. **B** Macroscopic images of representative B16F10*Luc2* metastatic livers of chow and CDAA groups (scale bars = 1 cm). **C** Counted hepatic metastasis (8 vs. 8, *p* = 0.0321, unpaired t-test); quantified metastatic percentage of whole liver area (5 vs. 7, *p* = 0.0130, unpaired t-test) and BLI measurement (photons/sec) (7 vs. 7, *p* = 0.0239, Mann–Whitney *U* test). **D** Total body weights (8 vs. 8, n.s., unpaired t-test), liver weights (8 vs. 8, *p* = 0.0193, unpaired t-test) and liver-to-body ratios (8 vs. 8, *p* = 0.0079, Mann–Whitney *U* test). **E** Setup for metastasis formation in CDAA diet-induced MASH model after intravenous injection (i.v.) of Wt31 cells and assessment of metastatic burden 19 days later. **F** Macroscopic images of representative Wt31 metastatic livers of chow and CDAA groups (scale bar = 1 cm). **G** Counted hepatic metastasis (6 vs. 5, *p* = 0.0045, Mann–Whitney *U* test), quantified metastatic percentage of whole liver area (6 vs. 5, *p* = 0.0027, unpaired t-test). **H** Total body weights (6 vs. 5, n.s., unpaired t-test), liver weights (6 vs. 5, *p* < 0.0001, unpaired t-test) and liver-to-body ratios (6 vs. 5, *p* = 0.0034, unpaired t-test)

To investigate hepatic melanoma metastasis in MASH, we analyzed fibrotic steatohepatitis in mice after 10 weeks of feeding a choline-deficient L-amino acid-defined (CDAA) diet. At week 10, B16F10*Luc2* melanoma cells were injected intrasplenically, and the CDAA diet was discontinued. The metastatic burden was assessed 14 days post-injection (Fig. 1A). Mice on the CDAA diet showed more liver metastases than the chow group (Fig. 1B), with significantly more metastatic lesions on the liver surface (Fig. 1C). ImageJ analysis revealed a greater metastatic surface area, and bioluminescence imaging (BLI) confirmed significantly higher signals from B16F10*Luc2* cells compared to the chow group (Fig. 1C). Liver weight and liver-to-body weight ratio increased in the CDAA group after 14 days, while body weight remained unchanged (Fig. 1D).

Wt31 melanoma cells, which grow more slowly than B16F10*Luc2* cells, were injected intravenously into mice fed a CDAA diet for 10 weeks. Metastatic burden was assessed 19 days after injection (Fig. 1E). Similar to B16F10*Luc2* results, CDAA-fed mice had significantly more Wt31 metastases and a larger metastatic surface area than the chow group (Fig. 1F-G). Liver weight and liver-to-body weight ratio were again increased, with no change in body weight (Fig. 1H).

### Continuation of the CDAA diet after melanoma cell seeding does not influence metastatic growth

To assess whether continued CDAA feeding after melanoma cell injection affected metastatic growth of B16F10*Luc2* and Wt31 cells, one group of mice remained on the CDAA diet, while another was switched to chow after injection (Supplemental Fig. 1A, 2A). A third control group was fed chow before and after cell injection. Both experimental groups (CDAA + and CDAA-) had similarly increased numbers of B16F10*Luc2* metastases and larger metastatic surface areas compared to controls (Supplemental Fig. 1B-D). Liver weight and liver-to-body ratio were also increased in CDAA + and CDAA- groups, while body weight remained unchanged (Supplemental Fig. 1E).

Similarly, intravenous Wt31 cell injection resulted in significantly more metastases in both CDAA + and CDAA- groups compared to controls, but no difference was observed between the two experimental groups (Supplemental Fig. 2B-C). Quantification of metastases confirmed these results (Supplemental Fig. 2D). Liver weight and liver-to-body ratio were significantly increased in both CDAA + and CDAA- groups, with no changes in body weight compared to controls (Supplemental Fig. 2E).

### Melanoma metastasis is increased in a genetic mouse model of MASH-like perisinusoidal liver fibrosis lacking hepatic steatosis

To investigate whether liver fibrosis without hepatic steatosis affects melanoma metastasis, we studied melanoma spread in genetically engineered *Gata4*^LSEC−KO^ mice, which develop MASH-like perisinusoidal liver fibrosis without steatosis due to a profibrotic switch in LSECs [14]. Notably, we could demonstrate in these previous experiments that 10 weeks of CDAA feeding significantly reduced GATA4 expression in wildtype LSECs accompanied by a similar transcriptomic shift in these wildtype LSECs as seen in *Gata4*^LSEC−KO^ mice.

**Fig. 2:**
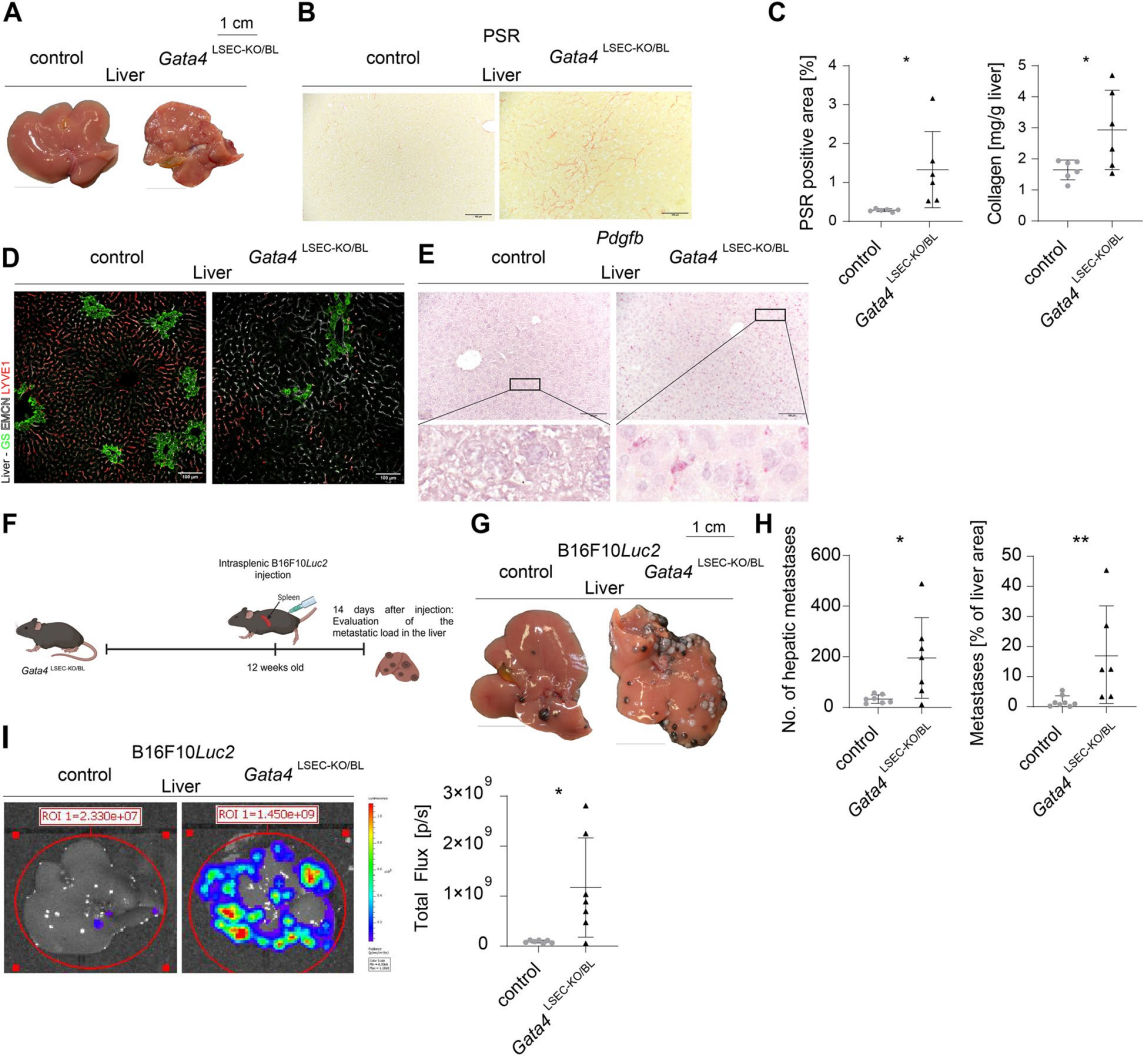
Endothelial *Gata4* deficiency causes hepatopathy and B16F10*Luc2* melanoma cells produce more hepatic metastasis in *Gata4*^LSEC−KO/BL^. **A** Macroscopic liver phenotype (scale bars = 1 cm). **B** Picrosirius Red (PSR) staining (scale bars = 100 µm). **C** Quantification of PSR positive area (6 vs. 6, *p* = 0.047, unpaired t-test) and hydroxyprolin assay in mg collagen/gram liver tissue (6 vs. 6, p = 0.0377, unpaired t-test). **D** Immunofluorescence staining of glutamine synthetase (GS), Endomucin (EMCN) and Lymphatic vessel endothelial hyaluronan receptor 1 (LYVE1) (scale bars = 100 µm). **E** In situ hybridization of *Pdgfb* in mouse liver (scale bars = 100 µm). **F** Setup for B16F10*Luc2* metastasis formation in *Gata4*^LSEC−KO/BL^ mice. Metastatic burden was assessed 14 days after intrasplenic B16F10*Luc2* injection. **G** Macroscopic images of representative B16F10*Luc2* metastatic livers (scale bars = 1 cm). **H** Counted hepatic metastasis (8 vs. 7, *p* = 0.0357, unpaired t-test); quantified metastatic percentage of whole liver area (8 vs. 6, *p* = 0.0080, Mann–Whitney U test). **I** Left panel: ex vivo BLI of livers 14 days after cell injection. Scale: Min: 6 × 10^6^ (p/sec/cm^2^/sr); Max: 1.1 × 10^8^ (p/sec/cm.^2^/sr). Livers were set as regions of interest and BLI signals were displayed. Right panel: measured BLI signal 14 days post injection (8 vs. 7, *p* = 0.0279, unpaired t-test)

*Gata4*^LSEC−KO^ mice were backcrossed to a C57BL/6 background (*Gata4*^LSEC−KO/BL^) and showed macroscopic liver fibrosis (Fig. 2A), increased hepatic collagen fibers (Fig. 2B-C), and reduced metabolic zonation (Fig. 2D, Supplemental Fig. 3A), while H&E staining did not show gross alterations (Supplemental Fig. 3B). The profibrotic angiocrine switch was confirmed by *Pdgfb* induction (Fig. 2E). Liver weight and liver-to-body ratio were significantly decreased, with body weight unchanged (Supplemental Fig. 3C).

For metastasis studies, B16F10*Luc2* cells were injected intrasplenically (Fig. 2F) and Wt31 cells intravenously (Supplemental Fig. 3D) into 12-week-old mice. *Gata4*^LSEC−KO/BL^ mice showed significantly increased liver metastases after injection of either cell line (Fig. 2G, Supplemental Fig. 3E), with quantification revealing a larger metastatic surface area (Fig. 2H, Supplemental Fig. 3F) and enhanced BLI signal (Fig. 2I). These results suggested that fully developed perisinusoidal liver fibrosis promotes hepatic melanoma metastasis independent of hepatic steatosis. Moreover, the prominent microvascular alterations in LSEC preceding perisinusoidal liver fibrosis in *Gata4*^LSEC−KO/BL^ mice [14] prompted us to further study whether early changes in the hepatic vascular niche might also contribute to enhanced hepatic melanoma metastasis in CDAA diet-induced steatohepatitis.

### Time course and sequence of pathogenic events in the development of CDAA diet-induced steatohepatitis

MASH progresses through several stages induced by multiple pathogenic hits that finally lead to liver fibrosis. In this stepwise process, vascular dysfunction is an early event characterized by a reduction of LSEC fenestrations occurring within one week of starting a CDAA diet. Reduction of LSEC fenestrations is paralleled by development of hepatic steatosis, but occurs well before the onset of liver fibrogenesis [21]. To identify optimal time points for studying metastasis at different stages of CDAA-induced steatohepatitis, we performed macroscopic and microscopic liver analyses at several time points (1 day, and 1, 2, 4, and 10 weeks).

Macroscopically, CDAA-fed mice showed larger, yellowish livers (Supplemental Fig. 4A), with no significant changes in body weight between CDAA and chow groups (Supplemental Fig. 4B). However, liver weight and liver-to-body weight ratio increased significantly starting from day one (Supplemental Fig. 4B). Microscopically, hepatic steatosis became evident at one week, as shown by H&E staining (Supplemental Fig. 4C), but PSR staining did not reveal collagen fibre deposition before 10 weeks (Supplemental Fig. 4D). Oil Red O staining detected increased lipid droplets from day one (Supplemental Fig. 4E), confirmed by quantification (Supplemental Fig. 4F). Plasma tests showed elevated alanine and aspartate aminotransferases at 1, 4, and 10 weeks, increased cholesterol at 1 day, 1, and 4 weeks, and elevated cholinesterase at multiple time points (Supplemental Fig. 4G). Plasma triglycerides tended to be reduced with prolonged periods of diet (Supplemental Fig. 4G).

### Diet-induced steatohepatitis promotes melanoma metastasis before development of overt MASH-associated perisinusoidal liver fibrosis

**Fig. 3:**
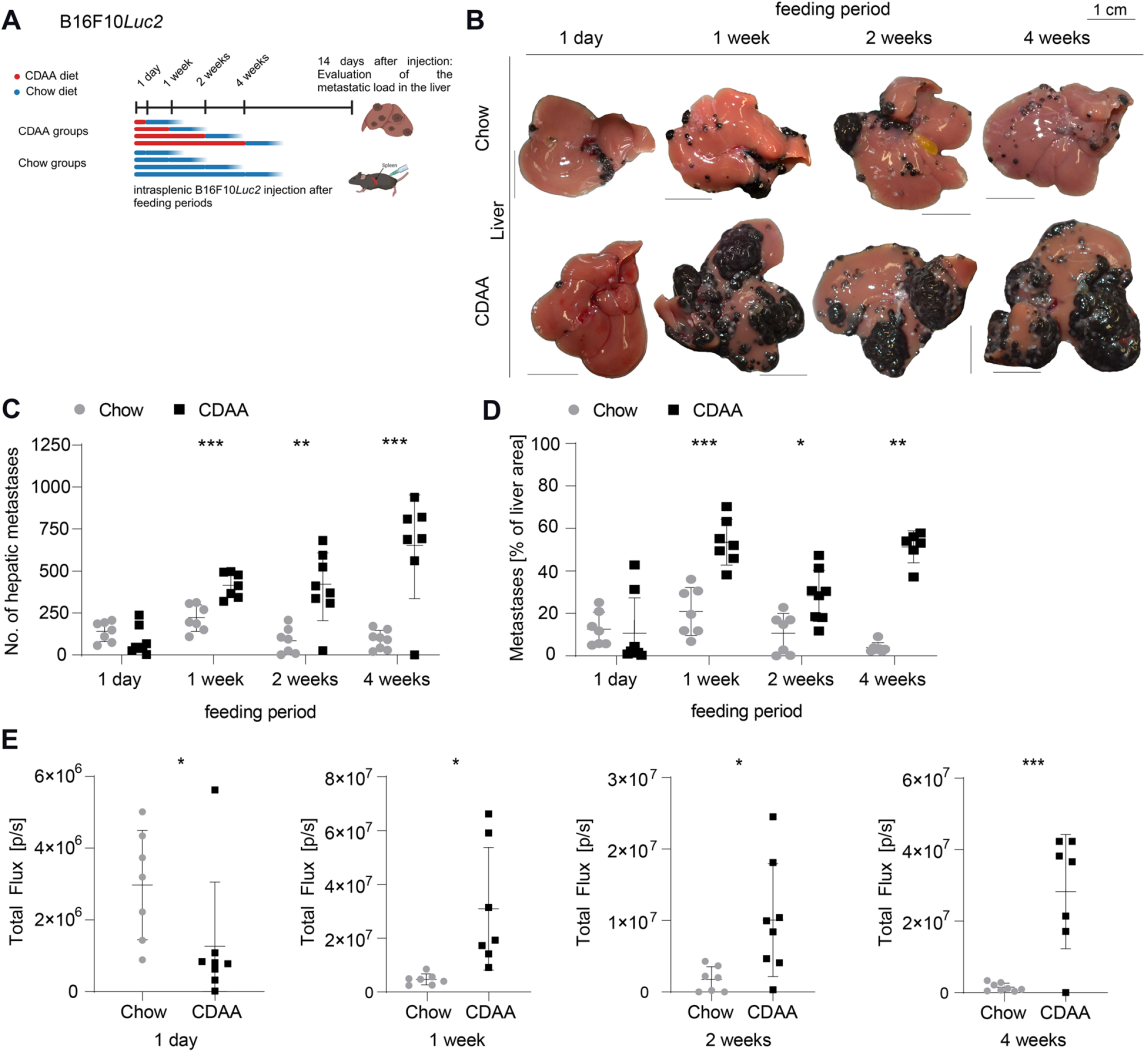
B16F10*Luc2* melanoma cells produce more hepatic metastasis after shorter CDAA feeding periods. **A** Setup for B16F10*Luc2* metastasis formation after shorter feeding periods of 1 day, 1, 2 and 4 weeks of CDAA diet. Metastatic burden was assessed 14 days after intrasplenic B16F10*Luc2* injection and discontinuation of CDAA diet. **B** Macroscopic images of representative B16F10*Luc2* metastatic livers of chow and CDAA groups (scale bars = 1 cm). **C** Counted hepatic metastasis (1 day, 7 vs. 8, n.s., Mann–Whitney *U* test; 1 week, 7 vs. 7, *p* = 0.0004, unpaired t-test; 2 weeks, 7 vs. 8, *p* = 0.009, unpaired t-test; 4 weeks, 8 vs. 7, *p* = 0.0002, unpaired t-test). **D** Quantified metastatic percentage of whole liver area (1 day, 7 vs. 8, n.s., Mann–Whitney *U* test; 1 week, 7 vs. 8, *p* = 0.0001, unpaired t-test; 2 weeks, 7 vs. 8, *p* = 0.0152, Mann–Whitney *U* test; 4 weeks, 7 vs. 6, *p* = 0.0012, Mann–Whitney *U* test). **E** Measured BLI signal 14 days post injection (1 day, 7 vs. 8, *p* = 0.0205, Mann–Whitney *U* test; 1 week, 8 vs. 7, *p* = 0.0225, unpaired t-test; 2 weeks, 7 vs. 8, *p* = 0.0294, unpaired t-test; 4 weeks, 8 vs. 7, *p* = 0.0004, unpaired t-test)

After demonstrating that fibrotic MASH promotes hepatic melanoma metastasis and observing early liver changes after 1 day of CDAA diet, we evaluated melanoma metastasis after 1 day, 1, 2, and 4 weeks of CDAA feeding. B16F10*Luc2* melanoma cells were injected intrasplenically at these time points, and the metastatic burden was analyzed 14 days later (Fig. 3A). No differences in liver metastases or metastatic surface area were detected after 1 day, with a reduced BLI signal (Fig. 3B-E). However, significantly higher numbers of metastases, larger metastatic areas, and enhanced BLI signals were observed after 1, 2, and 4 weeks of CDAA feeding (Fig. 3B-E), supported by quantification of intraparenchymal metastases (Supplemental Fig. 5A-B). Increased liver metastasis was also confirmed in male mice after 1 week of CDAA feeding (Supplemental Fig. 5C-D).

**Fig. 4:**
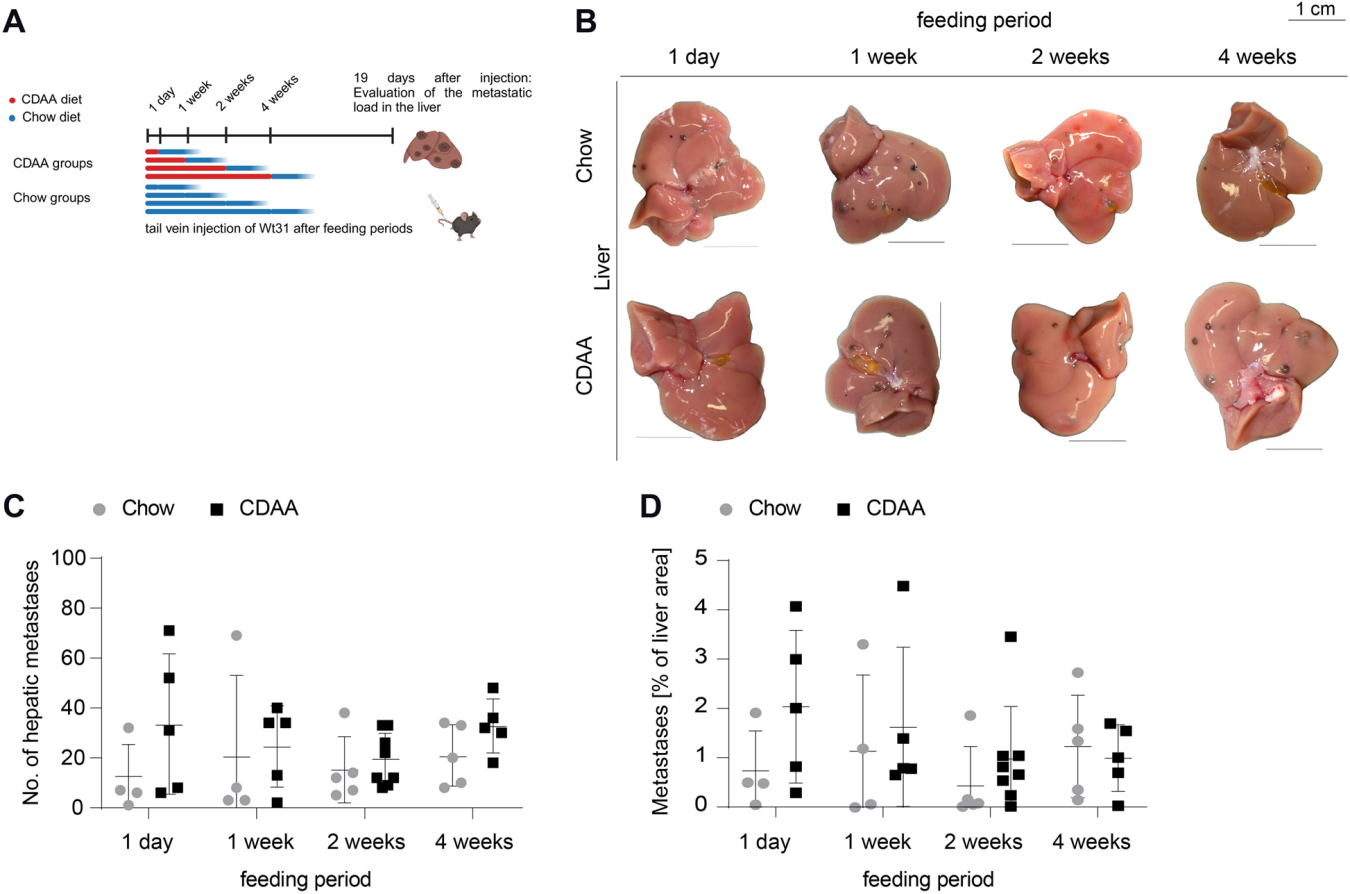
No differences in Wt31 hepatic metastasis formation metastasis after shorter CDAA feeding periods. **A** Setup for Wt31 metastasis formation after shorter feeding periods of 1 day, 1, 2 and 4 weeks of CDAA diet. Metastatic burden was assessed 19 days after intravenous Wt31 injection and discontinuation of CDAA diet. **B** Macroscopic images of representative Wt31 metastatic livers of chow and CDAA groups (scale bars = 1 cm). **C** Counted hepatic metastasis (1 day, 4 vs. 5, n.s., unpaired t-test; 1 week, 4 vs. 5, n.s., Mann–Whitney U test; 2 weeks, 5 vs. 8, n.s., unpaired t-test; 4 weeks, 5 vs. 5, n.s., unpaired t-test). **D** Quantified metastatic percentage of whole liver area (1 day, 5 vs. 6, n.s. unpaired t-test; 1 week, 4 vs. 5, n.s., Mann–Whitney U test; 2 weeks, 7 vs. 8, n.s., Mann–Whitney U test; 4 weeks, 5 vs. 5, n.s., unpaired t-test)

In parallel experiments with Wt31 melanoma cells injected intravenously after 1 day, 1, 2, and 4 weeks of CDAA feeding (Fig. 4A), no differences in the numbers of metastases or in metastatic areas were observed compared to chow-fed mice (Fig. 4B-D, Supplemental Fig. 5E-F), contrasting with the 10-week cohort (Fig. 1F-G). In order to exclude that the route of injection (intrasplenic versus intravenous) accounted for the differences in metastatic behaviour observed, Wt31 melanoma cells were also injected intrasplenically after 1 week of CDAA feeding (Supplemental Fig. 5G). Similar to intravenous injection of Wt31 cells, no significant differences in the numbers of metastases or in metastatic areas were observed compared to chow-fed mice (Supplemental Fig. 5H-I).

### High fat diet-induced hepatic steatosis is not sufficient to promote melanoma metastasis

To investigate whether hepatic steatosis without liver fibrosis affects melanoma metastasis, C57BL/6N mice were fed a high fat diet (HFD) for shorter periods of time, followed by intrasplenic injection of B16F10*Luc2* melanoma cells. The HFD contains over twice the crude fat (34.6%) compared to the CDAA diet (15%) [25], making it ideal for studying lipid influence on metastasis after shorter feeding periods. Before metastasis experiments, liver effects from shorter HFD were analyzed. No differences in body, liver, or liver-to-body ratio were observed between HFD-fed and chow-fed controls at 1 day, 1, 2, or 4 weeks (Supplemental Fig. 6A). H&E and PSR staining were unremarkable (Supplemental Fig. 6B-C), but ORO staining showed significantly enhanced lipid accumulation starting from day one (Supplemental Fig. 6D-E), with liver triglyceride levels being also significantly increased (Supplemental Fig. 6F). Liver function tests showed no differences in plasma triglycerides or liver enzymes, although plasma cholesterol levels were elevated (Supplemental Fig. 6G).

**Fig. 5:**
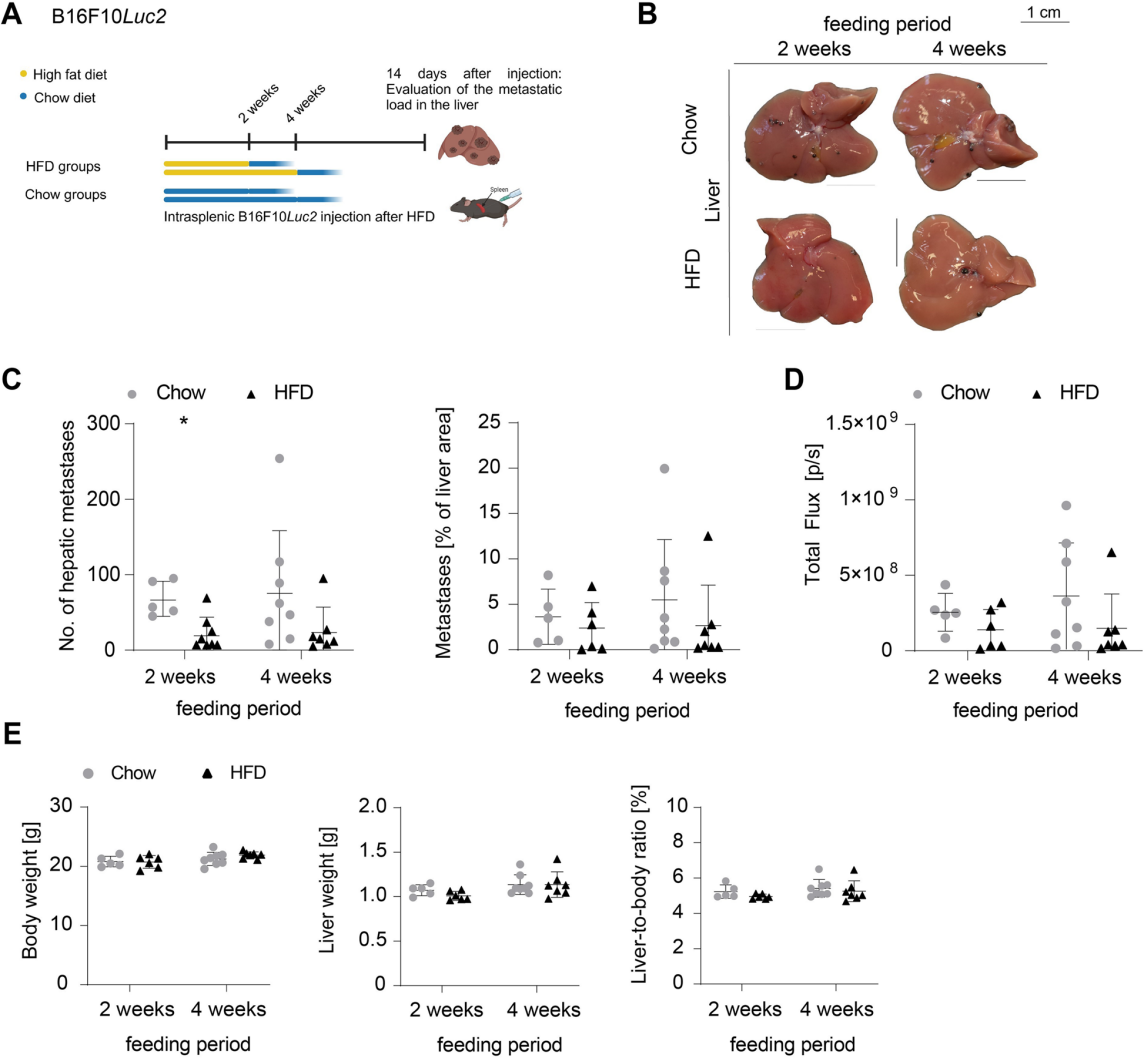
Enhanced metastasis formation is not observed after shorter high fat diet feeding periods. **A** Setup for B16F10*Luc2* metastasis formation after HFD feeding periods of 2 and 4 weeks. Metastatic burden was assessed 14 days after intrasplenic B16F10*Luc2* injection and discontinuation of HFD. **B** Macroscopic images of representative B16F10*Luc2* metastatic livers of chow and HFD groups (scale bars = 1 cm). **C** Counted hepatic metastasis (2 weeks, 5 vs. 6, *p* = 0.0303, unpaired t-test; 4 weeks, 8 vs. 7, n.s., Mann–Whitney *U* test); quantified metastatic percentage of whole liver area (2 weeks, 5 vs. 6, n.s., Mann–Whitney *U* test; 4 weeks, 8 vs. 7, n.s., Mann–Whitney *U* test). **D** Measured BLI signal 14 days post injection (2 weeks, 5 vs. 6, n.s., Mann–Whitney *U* test; 4 weeks, 8 vs. 7, n.s., Mann–Whitney *U* test). **E** Body weights, liver weights, and liver-to-body ratios (2 weeks, 5 vs. 6, n.s., unpaired t-test; 4 weeks, 8 vs. 7, n.s., unpaired t-test, 4 weeks liver weight: Mann–Whitney *U* test)

Melanoma metastasis was analyzed after 2 and 4 weeks of HFD (Fig. 5A). No significant differences in liver metastases or surface area were seen (Fig. 5B-C), although liver metastases were reduced after 2 weeks of HFD (Fig. 5C). BLI measurements and organ weight data (Fig. 5D-E) confirmed that hepatic steatosis alone did not promote melanoma metastasis, unlike the profibrotic MASH-like CDAA diet.

### Diet-induced steatohepatitis entails early inflammatory activation of the hepatic vascular niche

**Fig. 6:**
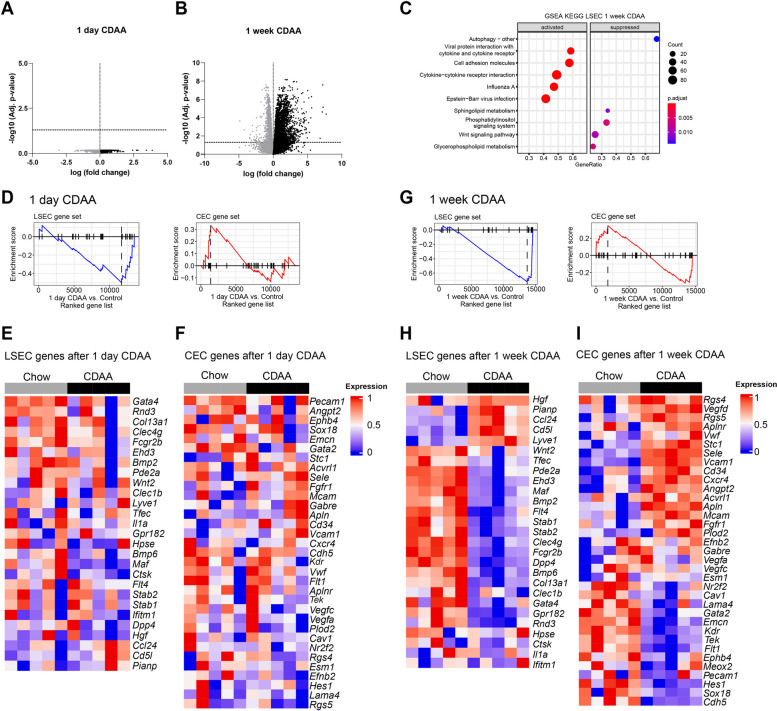
Transcriptomic analyses of isolated LSEC after 1 day and 1 week of CDAA diet (*n* = 5). **A** Volcano plot of all genes regulated after 1 day CDAA. Horizontal dashed line: significant cut off (-log(0.05)), vertical dotted line: log(fold change) = 0. Black dots are positive regulated genes, grey dots are negative regulated genes. **B** Volcano plot of all genes regulated after 1 week CDAA. Horizontal dashed line: significant cut off (-log(0.05)), vertical dotted line: log(fold change) = 0. Black dots are positively regulated genes, grey dots are negatively regulated genes. **C** GSEA KEGG pathway alterations analyzed using MSigDB hallmark gene sets in isolated LSEC after 1 week of CDAA diet. (**D-I**) LSEC and CEC associated genes are listed in Supplemental Table 1. **D** Enrichment plots of LSEC-associated (Normalized enrichment score (NES): −1,7; p.adjust: 0.026) and CEC-associated (NES: 1,2; p.adjust: 0.196) genes in isolated LSEC after 1 day of CDAA diet. **E** Graphical representation of the LSEC gene in a heat map after 1 day CDAA diet. The colour value indicates the expression level. Gene names are shown in italics. **F** Graphical representation of the CEC gene in a heat map after 1 day CDAA diet. The colour value indicates the expression level. Gene names are shown in italics. **G** Enrichment plots of LSEC-associated (Normalized enrichment score (NES): −2,4; p.adjust: 4,27 × 10^–07^) and CEC-associated (NES: 1,12; p.adjust:0,282) genes in isolated LSEC after 1 week of CDAA diet. **H** Graphical representation of the LSEC gene in a heat map after 1 week CDAA diet. The colour value indicates the expression level. Gene names are shown in italics. **I** Graphical representation of the CEC gene in a heat map after 1 week CDAA diet. The colour value indicates the expression level. Gene names are shown in italics

Bulk RNA-Seq was performed on LSECs from mice fed a CDAA diet for 1 day and 1 week compared to chow-fed controls to investigate changes in the hepatic vascular niche that might contribute to increased melanoma metastasis in early steatohepatitis. LSEC purity was analyzed by CD31 and LYVE1, markers of endothelial cells in general and of midzonal LSECs, respectively. The purity of LSECs isolated from chow-fed control animals was 95.1% (1 day) and 97.3% (1 week), while the purity of LSEC from mice fed a CDAA diet was 96.4% (1 day) and 87.4% (1 week). The lower purity of LSEC isolated from mice fed a CDAA diet for 1 week was mainly due to a higher percentage of contaminating CD11b^+^ myeloid cell populations (6,24% versus 0,36% CD11b^+^ cells in the CDAA-fed versus the chow-fed LSEC isolates, respectively) (Supplemental Fig. 7A). After 1 day of CDAA feeding, no genes reached significant changes (Fig. 6A), but after 1 week, many genes were significantly regulated (Fig. 6B). Gene Set Enrichment Analysis (GSEA) revealed dysregulated KEGG gene sets, including “cell adhesion molecules” (Fig. 6C). LSEC and continuous endothelial cell (CEC) gene sets showed a negative enrichment score for LSECs and a positive score for CECs after 1 day of CDAA feeding (Fig. 6D-F), and similar trends after 1 week (Fig. 6G-I). Whole liver RNA sequencing after 1 week of CDAA feeding revealed 153 dysregulated KEGG pathways, with 26 unique to LSECs and 68 unique to other liver cells (Supplemental Fig. 7B).

**Fig. 7:**
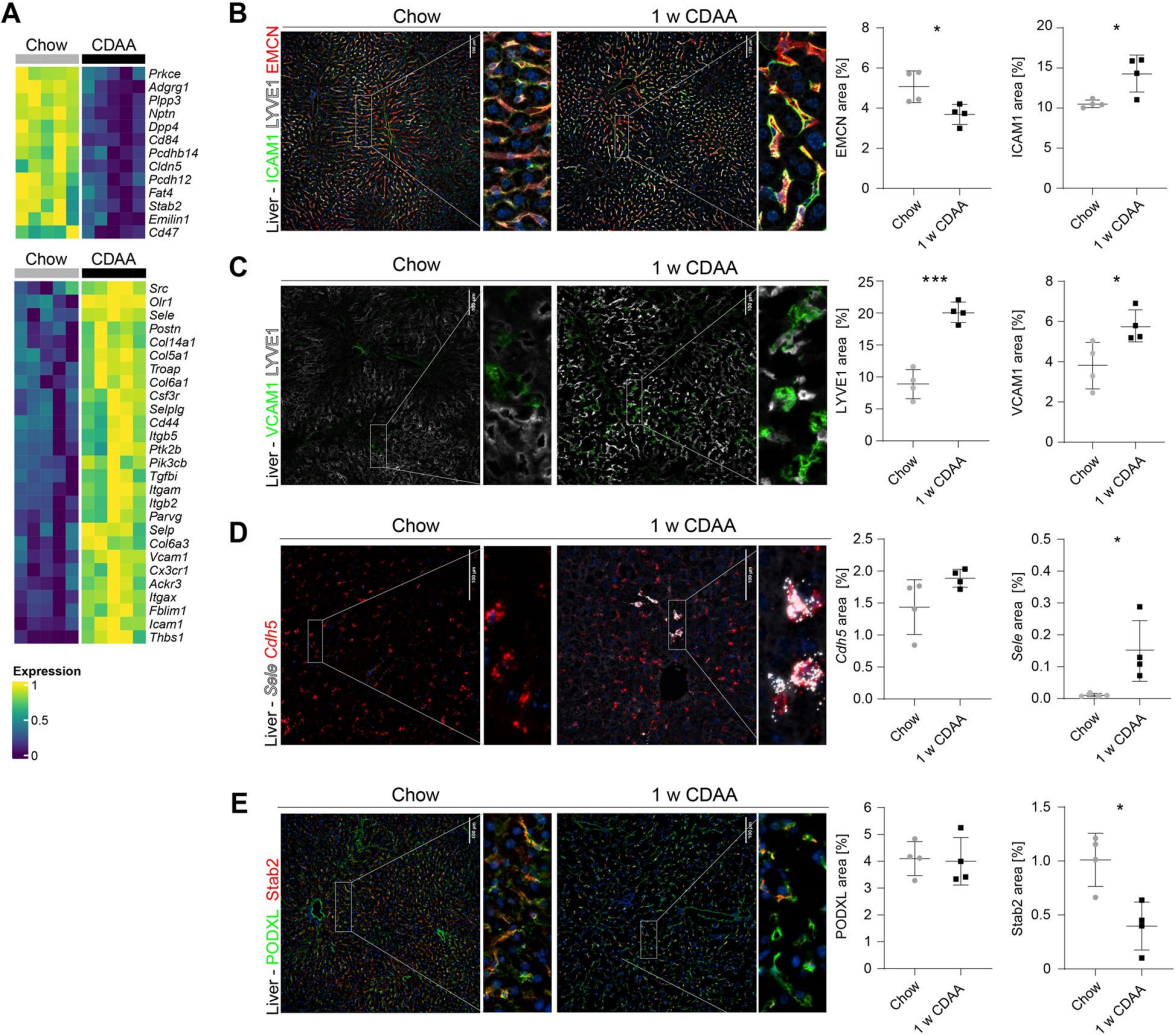
Endothelial cell adhesion molecules are upregulated during early phases of CDAA diet. **A** Heatmap of significantly 13 down- and 27 up-regulated genes in isolated LSEC after one week of CDAA diet (see Supplemental Table 2). **B**-**E** Immunofluorescence (IF) staining and fluorescence in situ hybridisation (FISH) (left side) and quantification (right side) of adhesion molecules and LSEC marker in mouse livers after 1 week of CDAA diet, *n* = 4, scale bars = 100 µm. **B** IF staining of ICAM1 (*p* = 0.0184, unpaired t-test), LYVE1 and EMCN (*p* = 0.0307, unpaired t-test). **C** IF staining of VCAM1 (*p* = 0.034, unpaired t-test) and LYVE1 (*p* = 0.003, unpaired t-test). **D** FISH staining of *Cdh5* (n.s., unpaired t-test) and *Sele* (*p* = 0.0273, unpaired t-test). **E** IF staining of PODXL (n.s., unpaired t-test) and Stab2 (*p* = 0.0103, unpaired t-test)

Focusing on adhesion molecules, 27 out of 40 DEGs were upregulated after 1 week, with *Vcam1*, *Icam1*, and *Sele* showing the highest significance (Fig. 7A, Supplemental Table 2). Immunofluorescence and in situ hybridisation confirmed endothelial-specific overexpression of these molecules (Fig. 7B-D) and the loss of the LSEC-specific marker Stab2 (Fig. 7E). No changes were observed after 1 day of CDAA feeding (Supplemental Fig. 7C), but increased expression especially of adhesion molecules was still seen after 4 and 10 weeks (Supplemental Fig. 7D-E). Beyond endothelial adhesion molecules, we analyzed the 30 most upregulated DEGs, with over one-third being macrophage-specific or macrophage-associated genes (Supplemental Fig. 8A). *Gpnmb* had the highest fold change among all DEGs (Supplemental Table 3). FISH co-staining of *Gpnmb* showed no colocalization with the endothelial marker *Cdh5* (Supplemental Fig. 8B), indicating *Gpnmb* induction in hepatic macrophages during early stages of CDAA diet. This was further confirmed by immunofluorescent staining of macrophage markers CD169 and CLEC4F, which were significantly upregulated in liver samples from mice fed the CDAA diet for 1 week (Supplemental Fig. 8C-D).

### Diet-induced steatohepatitis promotes melanoma cell retention in the hepatic vascular niche

**Fig. 8:**
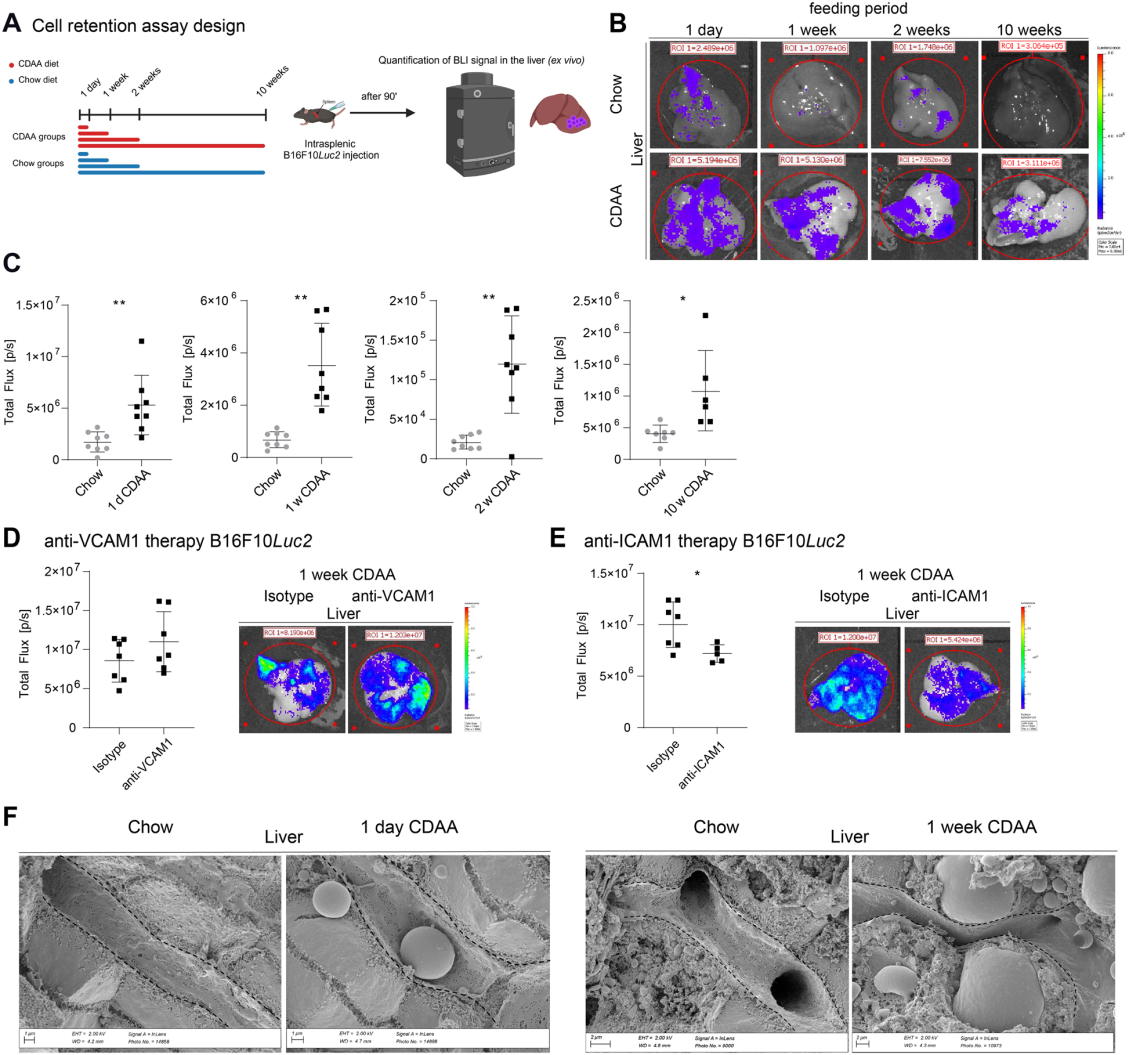
B16F10*Luc2* melanoma cells display a stronger intrahepatic retention after CDAA diet. **A** Setup for B16F10*Luc2* cell retention assay after 1 day, 1, 2 and 10 weeks of CDAA diet. **B** Ex vivo BLI of livers 90 min after cell injection. Scale: Min: 7 × 10^4^ (p/sec/cm^2^/sr); Max: 8 × 10^6^ (p/sec/cm^2^/sr). Livers were set as ROI and BLI signals were displayed. **C** Quantification of BLI signals in livers (1 day, 8 vs. 8, *p* = 0.0094, unpaired t-test; 1 week, 8 vs. 8, *p* = 0.0012, unpaired t-test; 2 weeks, 8 vs. 8, *p* = 0.0027, unpaired t-test; 10 weeks, 7 vs. 6, *p* = 0.0461, unpaired t-test). **D** Ex vivo BLI quantification and images of livers 90 min after cell injection and 24 h after anti-VCAM1 antibody therapy. Scale: Min: 7 × 10^4^ (p/sec/cm^2^/sr); Max: 1 × 10^6^ (p/sec/cm^2^/sr). Livers were set as ROI and BLI signals were displayed (7 vs. 7, *p* = 0.2836, unpaired t-test). **E** Ex vivo BLI quantification and images of livers 90 min after cell injection and 24 h after anti-ICAM1 antibody therapy. Scale: Min: 7 × 10^4^ (p/sec/cm^2^/sr); Max: 1 × 10^6^ (p/sec/cm^2^/sr). Livers were set as ROI and BLI signals were displayed (7 vs. 7, *p* = 0.0258, unpaired t-test). **F** Representative scanning electron micrographs of liver sinusoids from chow and 1 day (*n* = 3) and 1 week (*n* = 5) CDAA-fed mice. Dashed lines show sinusoidal vessel wall contour in Chow and CDAA groups. Scale bars = 1 or 2 µm

As LSECs in mice after 1 week of CDAA feeding showed strong induction of endothelial adhesion molecules, we hypothesized that melanoma cell adhesion to LSECs might initiate metastasis formation. To test this, we performed tumor cell retention experiments by injecting B16F10*Luc2* cells into the spleen and measuring hepatic BLI signals 90 min later (Fig. 8A). Increased BLI signals were observed in CDAA-fed mice after just one day and at longer feeding times (1, 2, and 10 weeks), indicating enhanced retention of B16F10*Luc2* cells (Fig. 8B-C). This was also confirmed in male mice after 1 week of CDAA feeding (Supplemental Fig. 9A). Similarly, Dil-labeled Wt31 melanoma cells showed enhanced retention in the liver upon intrasplenic injection after 1 week as well as upon intravenous injection after 2 weeks of CDAA feeding (Supplemental Fig. 9B-C). Anti-VCAM1 treatment showed no significant difference, but anti-ICAM1 treatment significantly reduced tumor cell retention of B16F10*Luc2* cells after 1 week of CDAA feeding (Fig. 8D-E).

Further analysis revealed sinusoidal occlusion with lipid droplets after 1 day of CDAA feeding and sinusoidal compression by lipid-laden hepatocytes after 1 week of CDAA feeding (Fig. 8F, Supplemental Fig. 9D-E). Doppler ultrasound showed an increased portal vein diameter and altered blood flow presumably representing a functional correlate of the mechanical compression of the sinusoids (Supplemental Fig. 9F). In addition, flow cytometry revealed that B16F10*Luc2* cells were larger than Wt31 cells (Supplemental Fig. 9G).

## Discussion

Recent advances in vascular biology have highlighted the active role of vascular endothelial cells (ECs) in various organ-specific functions [26]. ECs are now understood to be versatile cell populations that generate angiocrine stimuli and actively contribute to pathophysiological processes [27]. Here, we demonstrate that the hepatic vascular niche acts as a delicate sensor to even short-term nutritional alterations in the development of MASLD/MASH. The dynamic adaptations of the hepatic vascular niche in response to these metabolic challenges may be actively translated into aberrant functional programs, such as fibrotic regeneration and enhanced permissiveness for cancer metastasis.

In exploring the role of the altered hepatic vascular niche in metastasis, we analyzed the developmental steps of a murine model of MASH with late perisinusoidal liver fibrosis. The CDAA diet is now widely accepted as a relevant model for the study of MASH-associated liver fibrosis [14, 28]. Given that choline supplementation has been shown to improve endothelial cell senescence, proliferation, migration and angiogenesis in vitro [29], we expected that choline deficiency would not only alter liver homeostasis, but also have a significant impact on endothelial cell function. Notably, our observations revealed a gender-independent, significant increase in hepatic melanoma metastasis even during the early stages of MASH when perisinusoidal liver fibrosis was not yet developed. Conversely, hepatic melanoma metastasis was not enhanced in a mere model of incipient MASLD induced by feeding a high fat diet. Moreover, fully developed MASH-like perisinusoidal liver fibrosis without steatosis in *Gata4*^LSEC−KO/BL^ mice promoted hepatic melanoma metastasis; notably, perisinusoidal liver fibrosis in *Gata4*^LSEC−KO/BL^ mice is preceded by alterations in the hepatic microvascular bed including a pathogenic profibrotic angiocrine switch [14]. Altogether, these findings indicate that early pre-fibrotic changes in the hepatic vascular niche may be important in promoting enhanced melanoma metastasis to the liver. These findings differ from previous studies that have predominantly focused on later stages of MASH [16] or on toxic liver cirrhosis [30].

Intriguingly, we show here that early MASH induced by only one week of feeding a CDAA diet was characterized by prominent changes in the hepatic vascular niche. This is in line with the findings of Miyao et al. who identified early changes in LSECs under similar conditions [21]. Our study extends these observations by demonstrating dysregulation of LSEC-specific and of continuous endothelial cell genes as well as upregulation of cell adhesion molecules including VCAM1, ICAM1, and E-Selectin in LSECs. Expression of these adhesion molecules, especially ICAM1, might be directly involved in enhanced melanoma retention and hepatic metastasis as we demonstrate inhibition of melanoma cell retention by anti-ICAM1 antibody treatment. Alternatively, ICAM1 overexpression could influence various immune cell subsets, enhance their binding to the luminal surface of the sinusoids, and thus indirectly mediate melanoma cell retention and adhesion. Involvement of adhesion molecules in liver metastasis in general has been well documented. VCAM1 has been shown to facilitate metastatic invasion [31], and blocking E-Selectin reduced hepatic metastasis in H-59 carcinoma [32]. ICAM1 plays a key role in tumor cell adhesion to LSECs and aids their transmigration across endothelium [33]. While inhibition of NOTCH signalling increased lung metastasis in neuroblastoma and breast cancer [34], enhanced NOTCH signalling was reported by us to induce downregulation of endothelial ICAM1 in the liver accompanied by reduced hepatic melanoma metastasis [22].

The differences in melanoma metastasis and melanoma cell retention in the liver during the course of MASH development upon CDAA feeding and between the B16F10*Luc2* and Wt31 melanoma cell lines suggest a complex biology integrating melanoma cell-autonomous features (1), route of metastasis (2) as well as changing characteristics of the hepatic vascular niche including vascular alterations and inflammatory infiltrate (3).

Regarding melanoma cell-autonomous features, enhanced metastasis of B16F10*Luc2* cells compared to Wt31 cells may be due to their larger size, as demonstrated in this study, and to differential expression of integrins [23]. Although initial retention did not differ between B16F10*Luc2* cells and Wt31 cells, cell size may well be important as it may determine the time that melanoma cells are stuck in the sinusoids or the liver parenchyma, a longer time facilitating firm adhesion and thereby overt metastasis. This hypothesis would fit with our observation that Wt31 cells—in contrast to B16F10*Luc2* cells—do not exhibit enhanced metastasis after 1 week of CDAA feeding despite initially enhanced retention. Regarding expression of integrins by melanoma cell lines, our group has recently published a bulk RNA-Seq analysis of B16F10*Luc2* cells vs. Wt31 cells showing that integrins alpha 2b, −5, −7, −9 and −10, and beta 2 and −3 were significantly upregulated in B16F10*Luc2* cells as compared to Wt31 cells, while only Wt31 cells expressed integrin alpha4 [23]. Notably, liver-passaged B16 melanoma sublines have been reported to have increased integrin alpha2 expression and increased liver, but not lung metastasis [35]. On the contrary, integrin alpha4 is associated with lymph node metastasis [36]. In this regard, one could hypothesize that—besides cell size—the differences in integrin expression might explain why only B16F10*Luc2* cells form metastases after 1 week of CDAA feeding although both B16F10*Luc2* cells and Wt31 cells show enhanced initial retention.

In addition to cell-autonomous features, the route of injection may be important in explaining the differences in metastatic behavior and initial retention. Contact with the splenic or the lung parenchyma may influence differentiation of melanoma cells or cause formation of cellular complexes between tumor cells and circulating inflammatory cells, tumor cells and platelets, and tumor cells and soluble factors, e.g. coagulation factors, impacting on metastatic behavior. Our data, however, do not seem to support this hypothesis as we did not observe differences in metastasis between Wt31 cells injected intravenously or intrasplenically after 1 week of CDAA feeding.

Regarding changing features of the hepatic vascular niche during CDAA feeding, we observed that hepatic retention of B16F10*Luc2* cells was strong at all tested time intervals while we could not demonstrate enhanced melanoma metastasis after 1 day of CDAA feeding. In this respect, it has been shown that approximately 88% of melanoma cells injected into the liver are retained as solitary cells in the periportal sinusoids within 90 min of injection and 82% of these melanoma cells extravasate into the surrounding tissue by day 3 [37] indicating that further survival mechanisms beyond initial retention may be important for metastatic outgrowth in the liver. Notably, molecular alterations of LSEC were not yet to be detected after only 1 day of CDAA feeding including expression of ICAM1 and VCAM1. Therefore, we investigated whether CDAA feeding could support retention of melanoma cells mechanically. SEM revealed intravascular lipid droplets occluding the sinusoidal lumen after 1 day of CDAA feeding while lipid-laden hepatocytes compressed the sinusoidal lumen from the outside after 1 week of CDAA feeding. In addition, Doppler ultrasound showed changes in blood flow that could be caused by compression of sinusoidal lumina. As inhibition of enhanced ICAM1 expression in mice fed a CDAA diet for 1 week significantly reduced B16F10*Luc2* cell retention, we forward the hypothesis that mechanical retention followed by endothelial adhesion molecule- and integrin-mediated firm adhesion is necessary to permit definitive seeding of cancer cells to the premetastatic hepatic vascular niche prepared by early MASH.

Regarding the inflammatory infiltrate, macrophages also significantly impact the function of the hepatic vascular niche, especially during fibrosis [38]. As LSECs isolated from the livers of mice fed a CDAA diet for 1 week have a limited purity, especially regarding contamination by monocytes/macrophages, it was not surprising to find that bulk RNA-Seq of LSEC isolates showed 11 macrophage genes among the top differentially regulated genes. *Gpnmb* was the most highly upregulated macrophage gene in this analysis. GPNMB is known to be prevalent in Kupffer cells in MASH, and balances fibrosis and fibrolysis [39]. Furthermore, GPNMB is a potential cancer target, with high expression in most melanoma metastases [40]. Besides GPNMB, the numbers of CD169^+^ and CLEC4F^+^ macrophages were strongly enhanced in MASH livers of our CDAA fed mice. CD169^+^ macrophages exert differential effects, they mediate better outcomes and anti-metastatic actions in breast cancer [41, 42], but facilitate tumor immune escape via JAK2 signaling [43]. In addition, CLEC4F^+^ Kupffer cells are known to promote angiogenesis and tumor invasion [44]. Altogether, these findings suggest a complex role of monocytes/macrophages in the hepatic vascular niche in metastasis during MASLD/MASH progression.

## Conclusion

LSEC are increasingly recognized to play a critical role in organotropic liver metastasis [45]. In this study, we discovered that pathophysiological changes in the hepatic vascular niche, particularly in LSEC, are induced early on during the development of MASH which seem to be critical for the initial stages of melanoma metastasis formation in the liver. This early shift from the normal hepatic vascular niche towards a pre-metastatic hepatic vascular niche during the development of MASH becomes effective, however, only in a complex interaction with cell-autonomous features of circulating melanoma cells and the additional alterations likely acquired by them via the route of metastasis and the interaction with various organ environments and inflammatory infiltrates. Finally, our findings underline the importance of metabolic liver disease for metastasis and they may pave the way for novel angiotargeted therapeutic interventions.

## Supplementary Information

Additional file 1.

Additional file 2.

## Data Availability

The dataset(s) supporting the conclusions of this article is(are) included within the article (and its additional file(s)). Additional files: Supplemental Figures 1 – 9 are available as a PDF and include suppl. Figures 1 – 9 with legends. Supplemental Tables 1 – 3 are available in a word document and contain gene lists and expression values of mentioned genes. The datasets generated and analysed during the current study are available in the Gene Expression Omnibus database under the ac¬cession number GSE264249 [https://www.ncbi.nlm.nih.gov/geo/query/acc.cgi?acc=GSE264249], GSE264254 [https://www.ncbi.nlm.nih.gov/geo/query/acc.cgi?acc=GSE264254] and GSE264318 [https://www.ncbi.nlm.nih.gov/geo/query/acc.cgi?acc=GSE264318] and made publicly available. Data values for all graphs, and values behind any reported means in the manuscript or supplemental material are provided in the Supporting Data Values file.

## Abbreviations

ALT: Alanine transaminase
AST: Aspartate transaminase
BLI: Bioluminescence Imaging
CDAA: Choline-deficient L-amio acid-defined
CEC: Continuous endothelial cell
DEG: Differentially expressed genes
EMCN: Endomucin
FISH: Fluorescence in situ hybridization
FSC-A: Forward scatter area
FSC-H: Forward scatter height
FSC-W: Forward scatter width
h: Hour(s)
HFD: High Fat Diet
KEGG: Kyoto Encyclopedia of Genes and Genomes
LSEC: Liver sinusoidal endothelial cell
LYVE1: Lymphatic vessel endothelial hyaluronan receptor 1
MASH: Metabolic dysfunction-associated steatohepatitis
MASLD: Metabolic dysfunction-associated steatotic liver disease
NO: Nitric oxide
ORO: Oil Red O
PFA: Paraformaldehyde
PODXL: Podocalyxin
PSR: Picro-Sirius Red
RT: Room temperature
SSC-A: Sideward scatter area
Stab2: Stabilin-2
