# Supplementary material for: Steatohepatitis-induced vascular niche alterations promote melanoma metastasis
Source: Cancer Metab. 2025 Jan 28;13:5. doi: 10.1186/s40170-025-00374-6 (PMC11776123; doi:10.1186/s40170-025-00374-6)
Supplement: Supplementary file 1 — Additional file 1. [file 40170_2025_374_MOESM1_ESM.docx]

## Supplementary table 1. LSEC- and CEC-associated gene set.

| gs_name | genesymbol |
| --- | --- |
| lsec_genes | *Hpse* |
| lsec_genes | *Ehd3* |
| lsec_genes | *Bmp2* |
| lsec_genes | *Clec1b* |
| lsec_genes | *Ctsk* |
| lsec_genes | *Ifitm1* |
| lsec_genes | *Il1a* |
| lsec_genes | *Rnd3* |
| lsec_genes | *Maf* |
| lsec_genes | *Fcgr2b* |
| lsec_genes | *Flt4* |
| lsec_genes | *Pde2a* |
| lsec_genes | *Lyve1* |
| lsec_genes | *Tfec* |
| lsec_genes | *Ccl24* |
| lsec_genes | *Stab2* |
| lsec_genes | *Lmo3* |
| lsec_genes | *Cd5l* |
| lsec_genes | *Wnt2* |
| lsec_genes | *Pianp* |
| lsec_genes | *Gpr182* |
| lsec_genes | *Col13a1* |
| lsec_genes | *Dpp4* |
| lsec_genes | *Clec4g* |
| lsec_genes | *Gata4* |
| lsec_genes | *Hgf* |
| lsec_genes | *Bmp6* |
| lsec_genes | *Stab1* |
| cec_genes | *Pecam1* |
| cec_genes | *Cav1* |
| cec_genes | *Lama4* |
| cec_genes | *Fgfr1* |
| cec_genes | *Meox2* |
| cec_genes | *Emcn* |
| cec_genes | *Acvrl1* |
| cec_genes | *Sox18* |
| cec_genes | *Nr2f2* |
| cec_genes | *Hes1* |
| cec_genes | *Rgs5* |
| cec_genes | *Rgs4* |
| cec_genes | *Stc1* |
| cec_genes | *Plod2* |
| cec_genes | *Gabre* |
| cec_genes | *Cd34* |
| cec_genes | *Apln* |
| cec_genes | *Cxcr4* |
| cec_genes | *Vwf* |
| cec_genes | *Gata2* |
| cec_genes | *Wif1* |
| cec_genes | *Aplnr* |
| cec_genes | *Vegfa* |
| cec_genes | *Kdr* |
| cec_genes | *Tek* |
| cec_genes | *Cdh5* |
| cec_genes | *Sele* |
| cec_genes | *Esm1* |
| cec_genes | *Vegfc* |
| cec_genes | *Angpt2* |
| cec_genes | *Vcam1* |
| cec_genes | *Efnb2* |
| cec_genes | *Mcam* |
| cec_genes | *Vegfd* |
| cec_genes | *Flt1* |
| cec_genes | *Ephb4* |

## Supplementary table 2. Significantly differentially expressed genes (DEGs) in GO Cell adhesion pathway in CDAA diet fed mice compared to chow fed controls.

Shown are 40 significantly differentially regulated genes in LSECs from mice fed a CDAA diet for 1 week compared to LSECs from mice fed chow. Adjusted p-values were calculated for the differences of means of log2 of CDAA diet fed mice compared to chow controls. OneWay-ANOVA was performed to identify DEGs.

| **SYMBOL** | **Std1** | **Std2** | **Std3** | **Std4** | **Std5** | **CDAA1** | **CDAA2** | **CDAA3** | **CDAA4** | **CDAA5** | **logFC** | **adjP** |
| --- | --- | --- | --- | --- | --- | --- | --- | --- | --- | --- | --- | --- |
| ***Itgax*** | 3,123639 | 2,701219 | 2,814248 | 3,08512 | 2,256321 | 5,532584 | 5,82532 | 6,188347 | 5,654269 | 5,552765 | 2,951047 | 1,19E-08 |
| ***Plpp3*** | 11,64484 | 11,5406 | 11,50823 | 11,43769 | 11,54467 | 10,64263 | 10,47897 | 10,53277 | 10,69586 | 10,83226 | -0,91829 | 4,31E-08 |
| ***Vcam1*** | 6,857785 | 7,283185 | 6,981382 | 6,951201 | 6,694333 | 8,157989 | 8,158857 | 8,334989 | 8,207359 | 7,991497 | 1,279154 | 5,24E-08 |
| ***Nptn*** | 8,131002 | 8,104791 | 8,07371 | 8,104132 | 8,190511 | 7,470507 | 7,338218 | 7,397123 | 7,401924 | 7,522559 | -0,70927 | 5,24E-08 |
| ***Adgrg1*** | 8,392548 | 8,38077 | 8,234802 | 8,27892 | 8,337668 | 7,116458 | 7,163576 | 7,19137 | 7,326735 | 7,719582 | -1,06641 | 5,24E-08 |
| ***Ackr3*** | 4,802392 | 4,978364 | 5,413069 | 4,727071 | 4,593973 | 6,082912 | 6,54908 | 6,658534 | 6,323609 | 6,150049 | 1,564713 | 1,32E-07 |
| ***Icam1*** | 7,45769 | 7,29871 | 7,618216 | 7,409008 | 7,369966 | 8,381331 | 8,372252 | 8,584289 | 8,538857 | 8,278985 | 1,018672 | 3,91E-07 |
| ***Cldn5*** | 9,086659 | 8,800126 | 8,989347 | 9,022982 | 9,234803 | 8,305706 | 8,095902 | 8,156124 | 8,317797 | 8,281089 | -0,83945 | 4,6E-07 |
| ***Olr1*** | -1,80892 | -2,36248 | -5,20256 | -1,0313 | -3,9335 | 3,103089 | 3,047329 | 3,234662 | 2,969274 | 3,163795 | 5,585296 | 4,95E-07 |
| ***Sele*** | -0,0175 | 1,410114 | 0,947187 | 1,84602 | 1,020693 | 3,957892 | 4,187473 | 4,505207 | 4,090249 | 4,040976 | 3,136194 | 5,02E-07 |
| ***Cd84*** | 7,380329 | 7,423793 | 7,402224 | 7,254734 | 7,484526 | 6,60288 | 6,506759 | 6,648042 | 6,659829 | 6,620209 | -0,776 | 5,02E-07 |
| ***Itgb2*** | 5,207464 | 5,321689 | 5,03465 | 5,190815 | 4,510821 | 6,877175 | 7,392508 | 7,515343 | 6,539409 | 6,774234 | 2,036405 | 7,24E-07 |
| ***Parvg*** | 2,225354 | 2,473224 | 2,181144 | 2,67876 | 1,847856 | 4,637143 | 4,942788 | 5,341966 | 4,228854 | 4,371904 | 2,469303 | 9,54E-07 |
| ***Fblim1*** | 2,604386 | 2,204908 | 2,931863 | 2,527286 | 2,012916 | 4,342446 | 4,934952 | 4,825843 | 4,117698 | 4,466359 | 2,152955 | 1,72E-06 |
| ***Thbs1*** | 2,097974 | 2,380489 | 2,092061 | 2,096192 | 2,058963 | 3,698249 | 4,534572 | 4,555967 | 4,361056 | 4,030101 | 2,104849 | 1,99E-06 |
| ***Dpp4*** | 10,15044 | 10,27437 | 10,14385 | 10,04147 | 10,19919 | 9,568133 | 9,417453 | 9,450289 | 9,600836 | 9,639675 | -0,62181 | 2,04E-06 |
| ***Pcdh12*** | 9,458276 | 9,474851 | 9,186331 | 9,296551 | 9,407784 | 8,871995 | 8,675915 | 8,595402 | 8,834577 | 8,875733 | -0,6155 | 2,56E-06 |
| ***Col6a1*** | 2,714646 | 2,931124 | 2,504799 | 1,992035 | 2,229727 | 3,904097 | 4,187473 | 3,885605 | 4,219383 | 3,663366 | 1,586186 | 2,84E-06 |
| ***Col14a1*** | 4,182388 | 4,63125 | 4,014185 | 4,266914 | 4,169786 | 5,110743 | 5,412218 | 5,294926 | 5,52186 | 5,131092 | 1,044191 | 3,04E-06 |
| ***Col5a1*** | 2,817074 | 2,662354 | 1,538907 | 1,879769 | 2,058962 | 3,777321 | 4,130296 | 3,920889 | 4,040922 | 3,525628 | 1,652449 | 3,19E-06 |
| ***Tgfbi*** | 4,911657 | 5,160215 | 4,395493 | 4,994926 | 4,919286 | 5,864535 | 6,441895 | 6,54061 | 5,909181 | 5,977767 | 1,235026 | 3,52E-06 |
| ***Col6a3*** | 3,612179 | 4,260391 | 4,246587 | 3,760292 | 3,539526 | 4,7612 | 5,003984 | 4,941081 | 5,295718 | 5,231023 | 1,250905 | 3,73E-06 |
| ***Itgam*** | 3,086882 | 3,06594 | 2,448492 | 3,056165 | 2,563683 | 4,547249 | 4,876836 | 4,882155 | 4,167797 | 4,389027 | 1,713566 | 4,35E-06 |
| ***Postn*** | 1,177144 | 2,281381 | 0,863529 | 1,605011 | 2,028428 | 3,387003 | 3,787198 | 3,526996 | 4,235133 | 3,314803 | 2,004134 | 4,49E-06 |
| ***Pik3cb*** | 3,073422 | 3,097286 | 2,545633 | 2,931986 | 2,885062 | 4,101947 | 3,887333 | 4,346971 | 3,950238 | 4,177392 | 1,146739 | 4,9E-06 |
| ***Cx3cr1*** | 0,719463 | 1,54251 | 1,957311 | 1,291213 | 0,972872 | 3,520422 | 3,851044 | 4,149633 | 3,042715 | 3,536239 | 2,514076 | 7,32E-06 |
| ***Prkce*** | 7,029569 | 7,181227 | 7,101258 | 7,058238 | 7,063441 | 6,613691 | 6,289713 | 6,46825 | 6,591062 | 6,661899 | -0,55564 | 1,05E-05 |
| ***Emilin1*** | 9,273581 | 9,503511 | 9,117504 | 9,465663 | 9,518984 | 8,660074 | 8,67841 | 8,651622 | 9,016345 | 8,880408 | -0,64951 | 1,06E-05 |
| ***Troap*** | 1,925272 | 1,798516 | 1,667805 | 1,500423 | 0,748319 | 3,409243 | 3,674079 | 3,615809 | 3,873907 | 3,130244 | 2,087694 | 1,17E-05 |
| ***Cd47*** | 8,895295 | 9,047824 | 9,283066 | 8,965887 | 8,949227 | 8,499719 | 8,460736 | 8,37858 | 8,393732 | 8,683568 | -0,50727 | 1,27E-05 |
| ***Ptk2b*** | 4,343953 | 4,454862 | 4,009328 | 4,394967 | 3,750662 | 5,79379 | 5,921633 | 5,980171 | 4,930139 | 5,292984 | 1,454037 | 1,28E-05 |
| ***Stab2*** | 11,95384 | 11,94721 | 11,49042 | 11,71219 | 11,99026 | 11,16517 | 10,99006 | 11,14696 | 11,22391 | 11,3085 | -0,70153 | 1,37E-05 |
| ***Src*** | 1,690316 | 1,967446 | 2,836359 | 1,410925 | 2,243087 | 3,156655 | 3,433923 | 3,41597 | 3,112587 | 2,995117 | 1,36938 | 1,53E-05 |
| ***Pcdhb14*** | 4,558078 | 4,619852 | 4,55418 | 4,449633 | 4,721395 | 3,998796 | 3,900389 | 3,840565 | 4,20761 | 4,062482 | -0,5903 | 1,63E-05 |
| ***Itgb5*** | 5,40727 | 5,552975 | 5,18438 | 5,448659 | 4,668921 | 6,600469 | 7,064712 | 7,003902 | 6,183709 | 6,238522 | 1,44182 | 1,69E-05 |
| ***Fat4*** | 7,223023 | 7,275157 | 6,767905 | 7,130982 | 7,178719 | 6,329497 | 6,194463 | 6,251315 | 6,496277 | 6,713224 | -0,78654 | 1,7E-05 |
| ***Selp*** | 5,717655 | 5,865804 | 6,100648 | 6,078589 | 5,668272 | 6,373284 | 6,669658 | 6,634527 | 6,6534 | 6,716056 | 0,737189 | 1,92E-05 |
| ***Csf3r*** | 3,980492 | 3,698823 | 4,157882 | 4,119332 | 3,00901 | 5,872953 | 5,890576 | 6,150873 | 5,306193 | 5,568222 | 2,054441 | 1,97E-05 |
| ***Cd44*** | 5,310025 | 5,554399 | 4,87292 | 5,351279 | 4,022629 | 6,758768 | 7,461968 | 7,308497 | 6,08461 | 6,572916 | 1,912018 | 2,02E-05 |
| ***Selplg*** | 5,237998 | 4,884398 | 5,029861 | 5,460047 | 4,45164 | 6,506245 | 6,761127 | 6,825088 | 5,992173 | 6,160892 | 1,459295 | 2,08E-05 |

## Supplementary table 3. Significantly differentially expressed genes (DEGs) in CDAA diet fed mice compared to chow fed controls.

Shown are the top 30 upregulated DEGs in LSECs isolated from livers of mice fed CDAA for 1 week compared to LSECs from livers of chow fed mice. Adjusted p-values were calculated for differences in log2 means of CDAA-fed mice compared to chow controls. One-way ANOVA was used to identify DEGs.

| ***Gene Symbol*** | **Gene Title** | **logFC**  ***CDAA* > *chow*** | **Adjusted p-Value for Diff of experiment = *CDAA* - chow** |
| --- | --- | --- | --- |
| *Gpnmb* | glycoprotein (transmembrane) nmb | 7,7226 | 4,133E-05 |
| *Vsig8* | V-set and immunoglobulin domain containing 8 | 7,2860 | 7,191E-06 |
| *Mmp12* | matrix metallopeptidase 12 | 7,2330 | 2,608E-08 |
| *Gdf15* | growth differentiation factor 15 | 7,1624 | 2,390E-05 |
| *Mup20* | major urinary protein 20 | 6,8405 | 1,779E-02 |
| *Dcstamp* | dendrocyte expressed seven transmembrane protein | 5,6155 | 8,486E-06 |
| *Olr1* | oxidized low density lipoprotein (lectin-like) receptor 1 | 5,5853 | 4,946E-07 |
| *Gdf3* | growth differentiation factor 3 | 5,4846 | 1,045E-04 |
| *Gm4294* | ribosomal protein L15 pseudogene | 5,3065 | 3,992E-03 |
| *Ccl9* | chemokine (C-C motif) ligand 9 | 5,1977 | 6,897E-07 |
| *Gm21188* | predicted gene, 21188 | 5,1707 | 3,578E-04 |
| *Il1rn* | interleukin 1 receptor antagonist | 5,0228 | 1,116E-07 |
| *H2ac12* | H2A clustered histone 12 | 4,9668 | 1,592E-02 |
| *Clec4d* | C-type lectin domain family 4, member d | 4,8819 | 4,381E-03 |
| *Acod1* | aconitate decarboxylase 1 | 4,8331 | 2,368E-05 |
| *Cxcl3* | chemokine (C-X-C motif) ligand 3 | 4,8225 | 1,531E-03 |
| *Ocstamp* | osteoclast stimulatory transmembrane protein | 4,8191 | 1,816E-04 |
| *Gm6851* | protein (peptidyl-prolyl cis/trans isomerase) NIMA-interacting, 4 (parvulin) pseudogene | 4,7758 | 4,806E-03 |
| *Lama2* | laminin, alpha 2 | 4,6464 | 1,150E-03 |
| *Tnc* | tenascin C | 4,6160 | 9,477E-05 |
| *Gpr84* | G protein-coupled receptor 84 | 4,6002 | 2,152E-04 |
| *Trem1* | triggering receptor expressed on myeloid cells 1 | 4,5992 | 2,059E-03 |
| *Siglecf* | sialic acid binding Ig-like lectin F | 4,5247 | 3,736E-05 |
| *Ssc5d* | scavenger receptor cysteine rich family, 5 domains | 4,5220 | 2,602E-04 |
| *Nkain1* | Na+/K+ transporting ATPase interacting 1 | 4,5101 | 1,704E-04 |
| *Mmp13* | matrix metallopeptidase 13 | 4,4958 | 1,941E-06 |
| *Fut7* | fucosyltransferase 7 | 4,4791 | 3,768E-04 |
| *Clec5a* | C-type lectin domain family 5, member a | 4,4075 | 1,306E-04 |
