# Supplementary material for: Steatohepatitis-induced vascular niche alterations promote melanoma metastasis
Source: Cancer Metab. 2025 Jan 28;13:5. doi: 10.1186/s40170-025-00374-6 (PMC11776123; doi:10.1186/s40170-025-00374-6)
Supplement: Supplementary file 2 — Additional file 2. [file 40170_2025_374_MOESM2_ESM.docx]

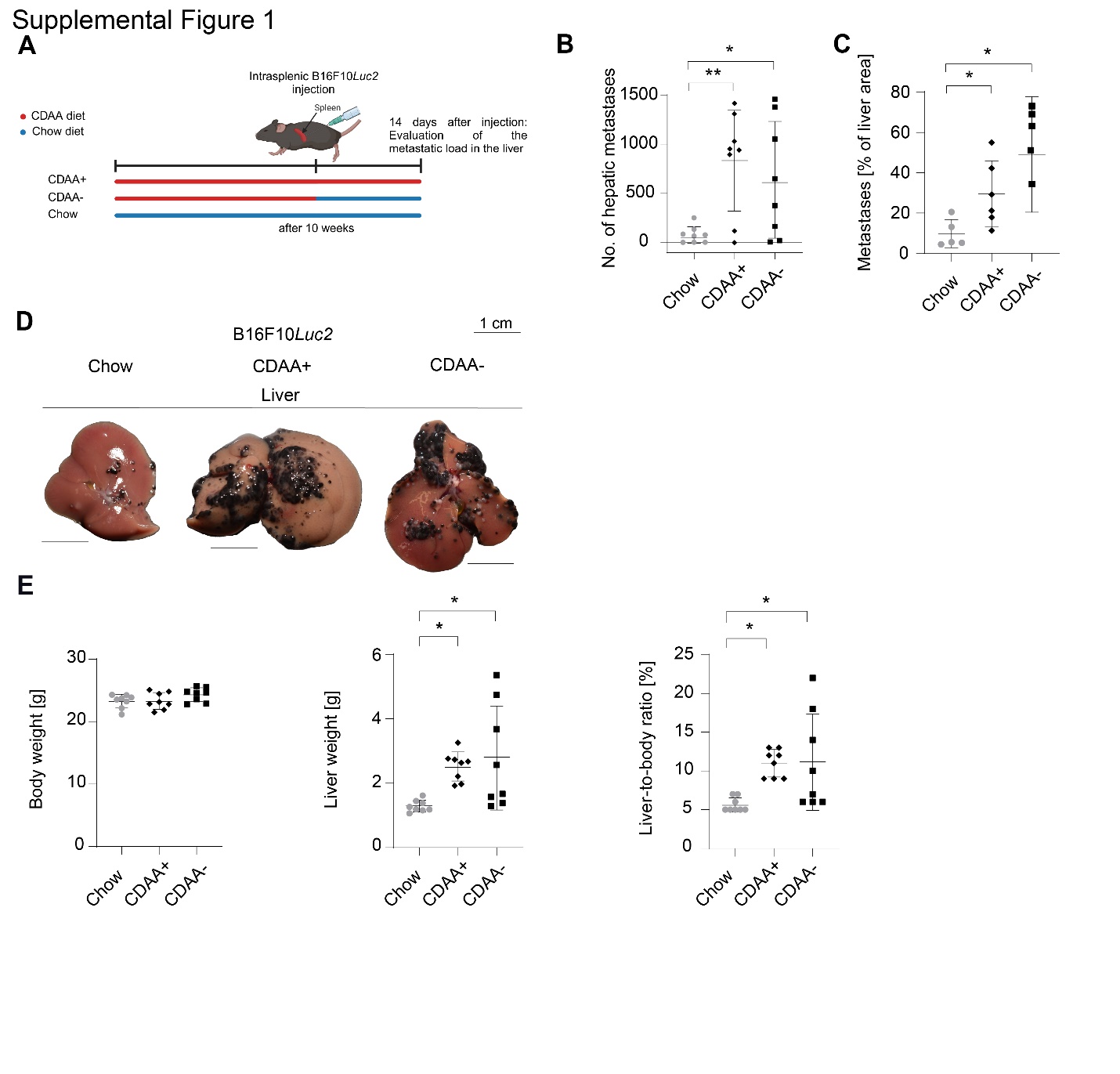
**Supplemental Figure 1. Assessment of** **B16F10*Luc2* hepatic metastatic formation after continuation and discontinuation of CDAA diet. (A)** Experimental setup for metastasis formation in CDAA diet-induced MASH model after intrasplenic injection of B16F10*Luc2* cells. Assessment of metastatic burden 14 days post injections during both continuation (CDAA+) and discontinuation (CDAA-) of CDAA diet. **(B)** Counted hepatic metastasis numbers (8 chow vs. 8 CDAA+ vs. 8 CDAA-, p=0.0091, One-Way ANOVA). **(C)** Quantified metastatic percentage of whole liver area (5 vs. 6 vs. 5, p=0.0219, One-Way ANOVA). **(D)** Macroscopic images of representative B16F10*Luc2* metastatic livers of chow and CDAA groups (scale bars=1 cm). **(E)** Total body weights (8 vs. 8 vs. 8, n.s., One-Way ANOVA), liver weights (8 vs. 8 vs. 8, p=0.0131, One-Way ANOVA) and liver-to-body ratios (8 vs. 8 vs. 8, p=0.0117, One-Way ANOVA).

**
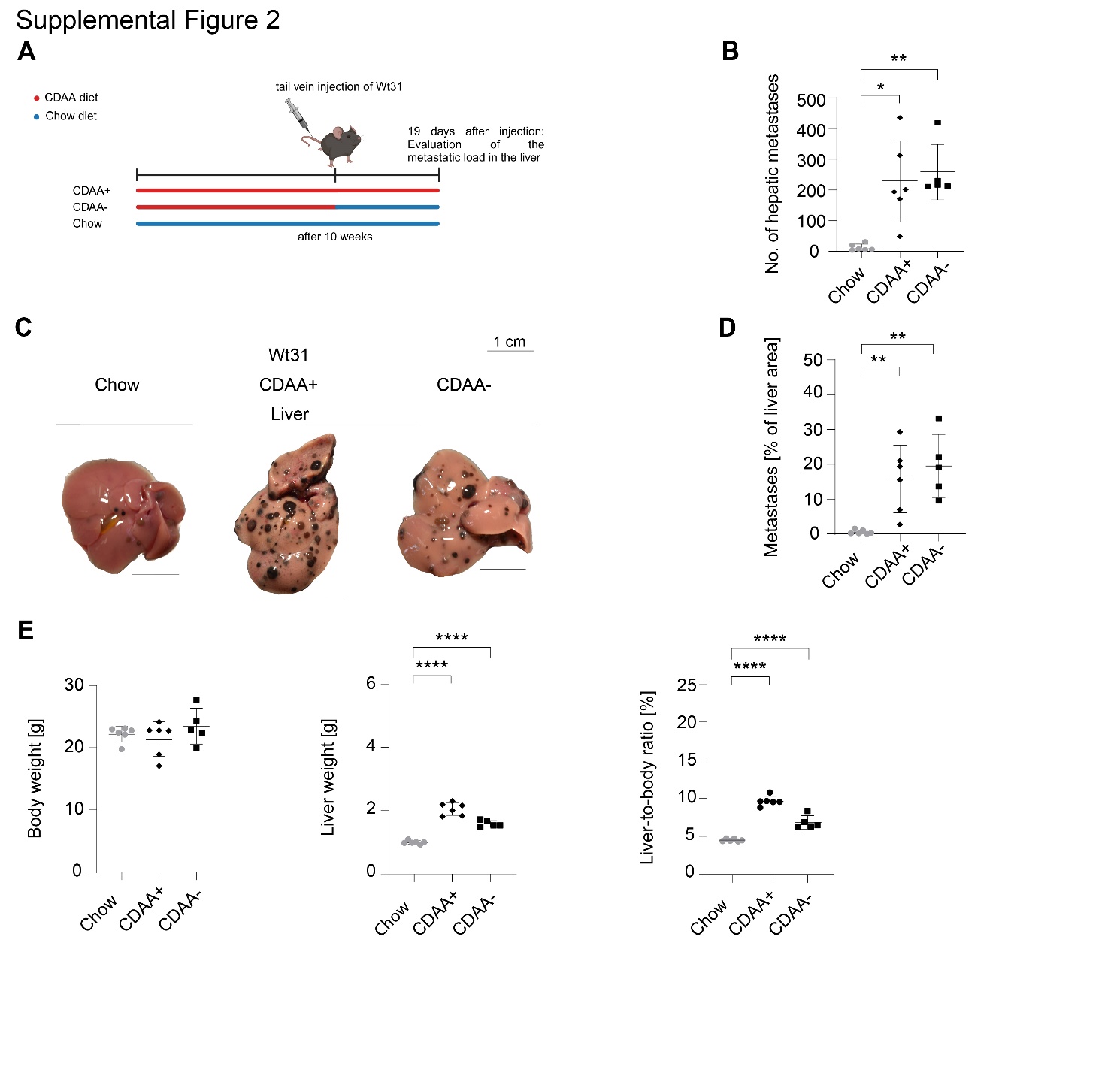
Supplemental Figure 2. Assessment of** **Wt31 hepatic metastatic formation after continuation and discontinuation of CDAA diet. (A)** Experimental setup for metastasis formation in CDAA diet-induced MASH model after intravenous injection of Wt31 cells. Assessment of metastatic burden 19 days post injections during both continuation (CDAA+) and discontinuation (CDAA-) of CDAA diet. **(B)** Counted hepatic metastasis numbers (6 vs. 6 vs. 5, p=0.0009, One-Way ANOVA). **(C)** Macroscopic images of representative Wt31 metastatic livers of chow and CDAA groups (scale bars=1 cm). **(D)** Quantified metastatic percentage of whole liver area (6 vs. 6 vs. 5, p=0.002, One-Way ANOVA). **(E)** Total body weights (6 vs. 6 vs. 5, n.s., One-Way ANOVA), liver weights (6 vs. 6 vs. 5, p<0.0001, One-Way ANOVA) and liver-to-body ratios (6 vs. 6 vs. 5, p<0.0001, One-Way ANOVA).

**
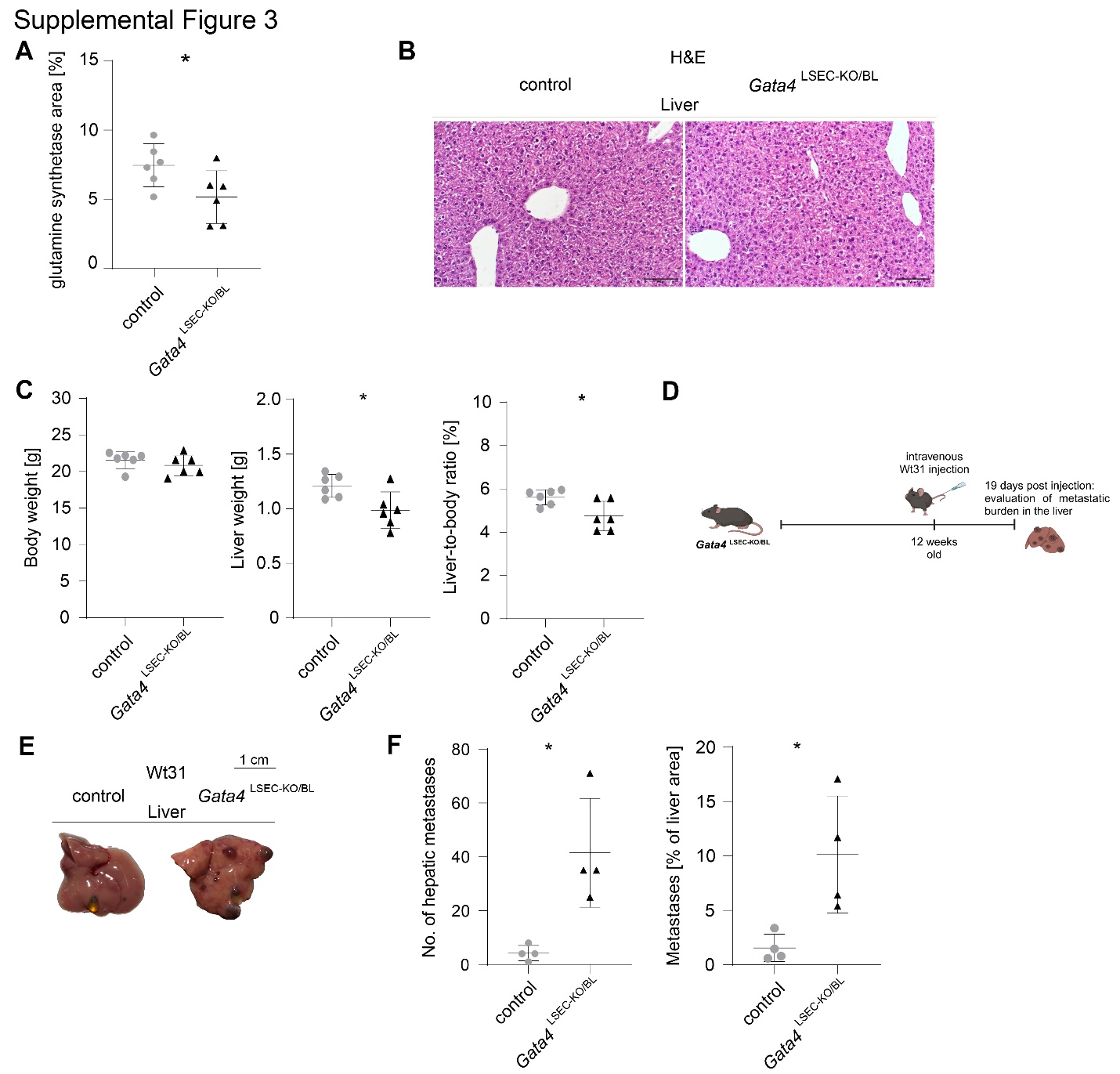
Supplemental Figure 3. Hepatic characterization of LSEC specific *Gata4* knock out in mice. (A)**Quantification of glutamine synthetase positive area in livers of control and *Gata4*^LSEC-KO/BL^ mice (n=6, p=0.448, unpaired t-test). **(B)** Histopathological images with H&E staining of *Gata4*^LSEC-KO/BL^ and control mice (scale bars=100 µm). **(C)** Total body weights (6 vs. 6, n.s., unpaired t-test), liver weights (6 vs. 6, p=0.0198, unpaired t-test), liver-to-body ratios (6 vs. 6, p=0.0204, unpaired t-test) of *Gata4*^LSEC-KO/BL^ and control mice. **(D)** Setup for Wt31 metastasis formation in *Gata4*^LSEC-KO/BL^ mice. Metastatic burden was assessed 19 days after intravenous Wt31injection. **(E)** Macroscopic images of representative Wt31 metastatic livers (scale bars=1 cm). **(F)** Counted hepatic metastasis (4 vs. 4, p=0.0333, unpaired t-test); quantified metastatic percentage of whole liver area (4 vs. 4, p=0.0463, unpaired t-test).

**
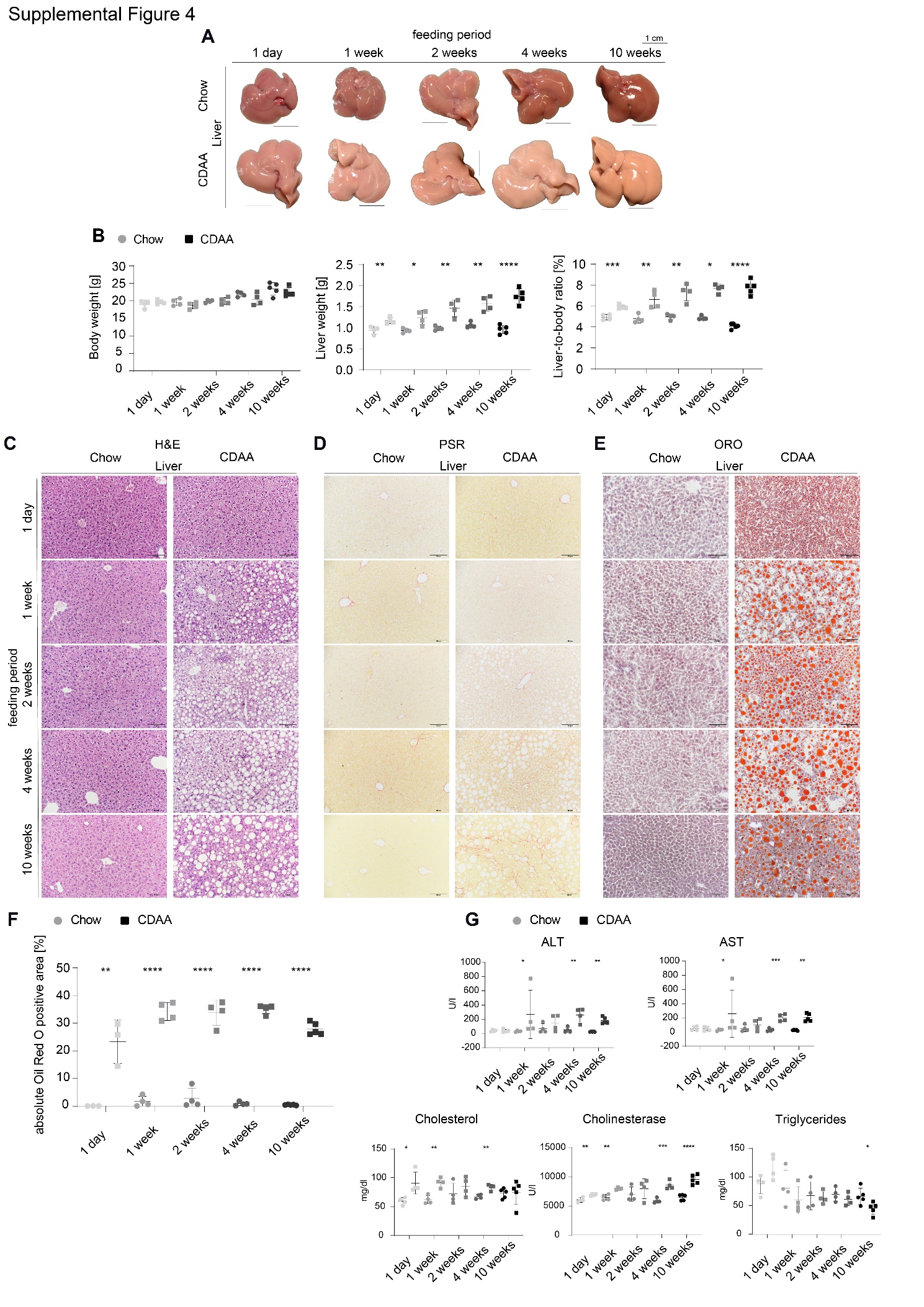
Supplemental Figure 4. Time series of liver characterisation at different CDAA feeding durations. (A)** Macroscopic images of representative livers of chow and CDAA groups (scale bars=1 cm). **(B)** Total body weights, liver weights and liver-to-body ratios during feeding periods of 1 day, 1, 2 and 4 weeks with n=4 and 10 weeks with n=5 per group (liver weights: 1 day, p=0.0086, unpaired t-test; 1 week, p=0.0129, unpaired t-test; 2 weeks, p=0.0037, unpaired t-test; 4 weeks, p=0.0017, 10 weeks, p<0,0001, unpaired t-test and liver-to-body ratios: 1 day, p=0.0010, unpaired t-test; 1 week, p=0.0064, unpaired t-test; 2 weeks, p=0.0016, unpaired t-test; 4 weeks, p=0.0286, Mann-Whitney *U* test; 10 weeks, p<0,0001, unpaired t-test; body weight differences are statistically not significant (unpaired t-tests). **(C-E)** Histopathological images of livers from chow and CDAA-fed mice with **(C)** Hematoxylin and eosin (H&E) staining (scale bars=100 µm), **(D)** Picrosirius Red (PSR) staining (scale bars=100 µm) and **(E)** Oil Red O (ORO) staining (scale bars=100 µm). **(F)** Quantification of ORO positive percentage of whole liver area (1 day, p=0.0069, unpaired t-test; 1 week, p<0.0001, unpaired t-test; 2 weeks, p<0.0001, unpaired t-test; 4 weeks, p<0.0001, unpaired t-test; 10 weeks, p<0.0001, unpaired t-test). **(G)** Plasma levels of alanine aminotransferase (ALT) (1 day, n.s., unpaired t-test; 1 week, p=0.0286, Mann-Whitney *U* test; 2 weeks, n.s., unpaired t-test; 4 weeks, p=0,0079, unpaired t-test; 10 weeks, p=0,0079, Mann-Whitney *U* test ), aspartate aminotransferase (AST) (1 day, n.s., unpaired t-test; 1 week, p=0.0286, Mann-Whitney *U* test; 2 weeks, n.s., unpaired t-test; 4 weeks, p=0,0007, unpaired t-test; 10 weeks, p=0,0015, unpaired t-test), total cholesterol (1 day, p=0,0286, Mann-Whitney *U* test; 1 week, p=0.0017, unpaired t-test; 2 weeks, n.s., unpaired t-test; 4 weeks, p=0,0024, unpaired t-test; 10 weeks, n.s., unpaired t-test), cholinesterase (1 day, p=0,006, unpaired t-test; 1 week, p=0.0014, unpaired t-test; 2 weeks, n.s. , unpaired t-test; 4 weeks, p=0,0008, unpaired t-test; 10 weeks, p<0.0001, unpaired t-test) and triglycerides (1 day, n.s., unpaired t-test; 1 week, n.s., unpaired t-test; 2 weeks, n.s., unpaired t-test; 4 weeks, n.s., unpaired t-test; 10 weeks, p=0.0276, unpaired t-test) in control mice and CDAA-fed mice, n=4.

**
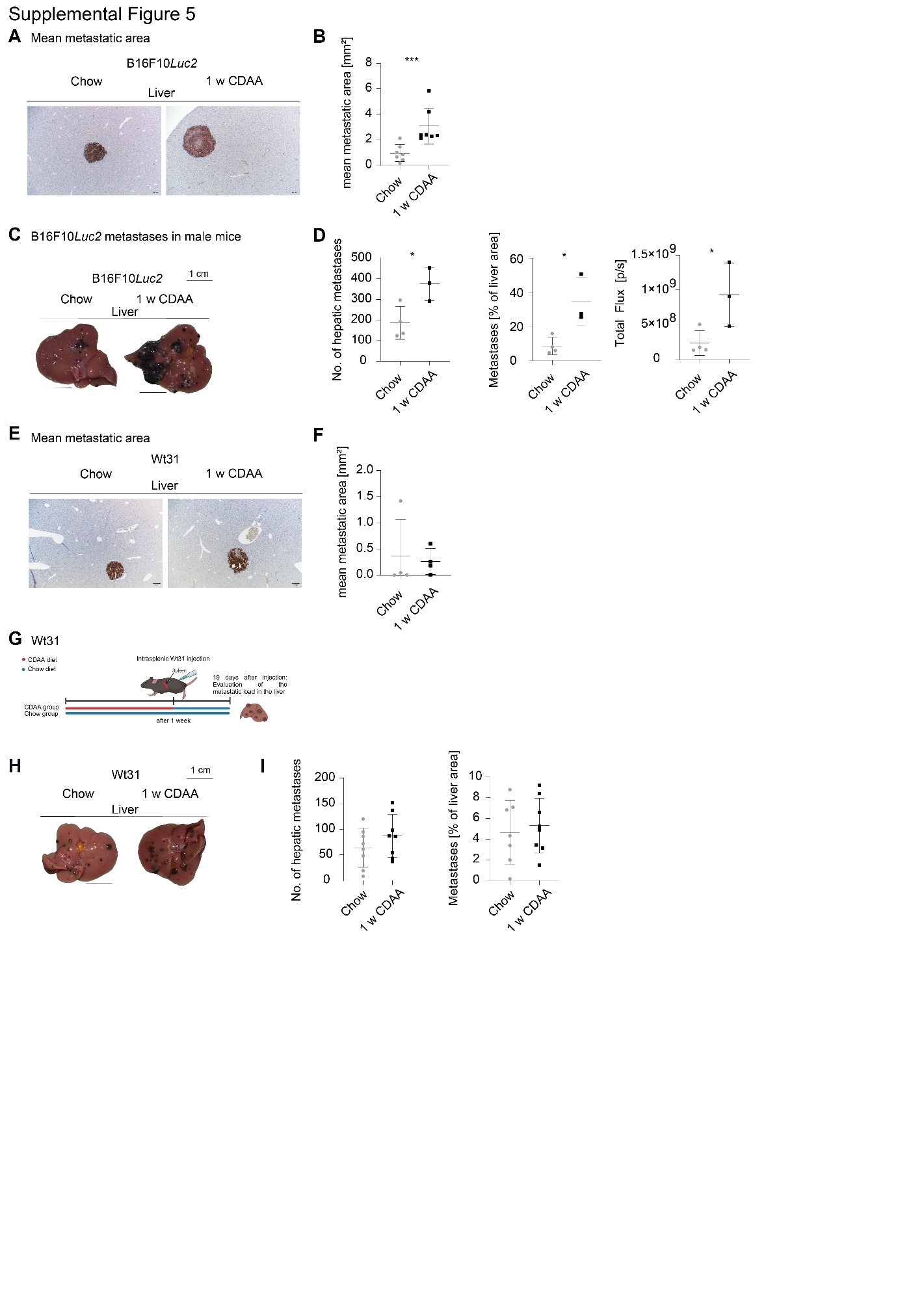
Supplemental Figure 5. Additional metastasis experiments and further quantifications to validate the previously observed findings. (A)** Microscopic images of immunohistochemical TRP-2 staining representing hepatic B16F10*Luc2* metastases from the chow and CDAA groups (scale bars=200µm). **(B)** Quantified mean metastatic area of TRP-2-positive B16F10*Luc2* metastases in liver sections (7 vs. 7, p=0.0006, Mann-Whitney *U* test). **(C)** Macroscopic images of representative B16F10*Luc2* metastatic livers of chow and CDAA groups in male mice (scale bars=1 cm). **(D)** Counted hepatic B16F10*Luc2* metastases numbers (4 vs. 3, p=0.0273, unpaired t-test); quantified metastatic percentage of whole liver area (4 vs. 3, p=0.0176, unpaired t-test) and BLI photon flux measurement (photons per sec) in male mice (4 vs. 3, p=0.0374, unpaired t-test). **(E)** Microscopic images of immunohistochemical TRP-2 staining representing hepatic Wt31 metastases from the chow and CDAA groups (scale bars=200µm). **(F)** Quantified mean metastatic area of TRP-2-positive Wt31 metastases in liver sections (4 vs. 4, n.s., Mann-Whitney *U* test). (**G**) Experimental setup for Wt31 metastases formation after intrasplenic injection. Metastatic burden was assessed 19 days after intrasplenic injection of Wt31 and discontinuation of the 1-week CDAA diet. (**H**) Macroscopic images of representative Wt31 metastatic livers of chow and CDAA groups (scale bars=1 cm). (**I**) Counted hepatic Wt31 metastases numbers (8 vs. 8, n.s., unpaired t-test); quantified metastatic percentage of whole liver area (8 vs. 8, n.s., unpaired t-test).

**
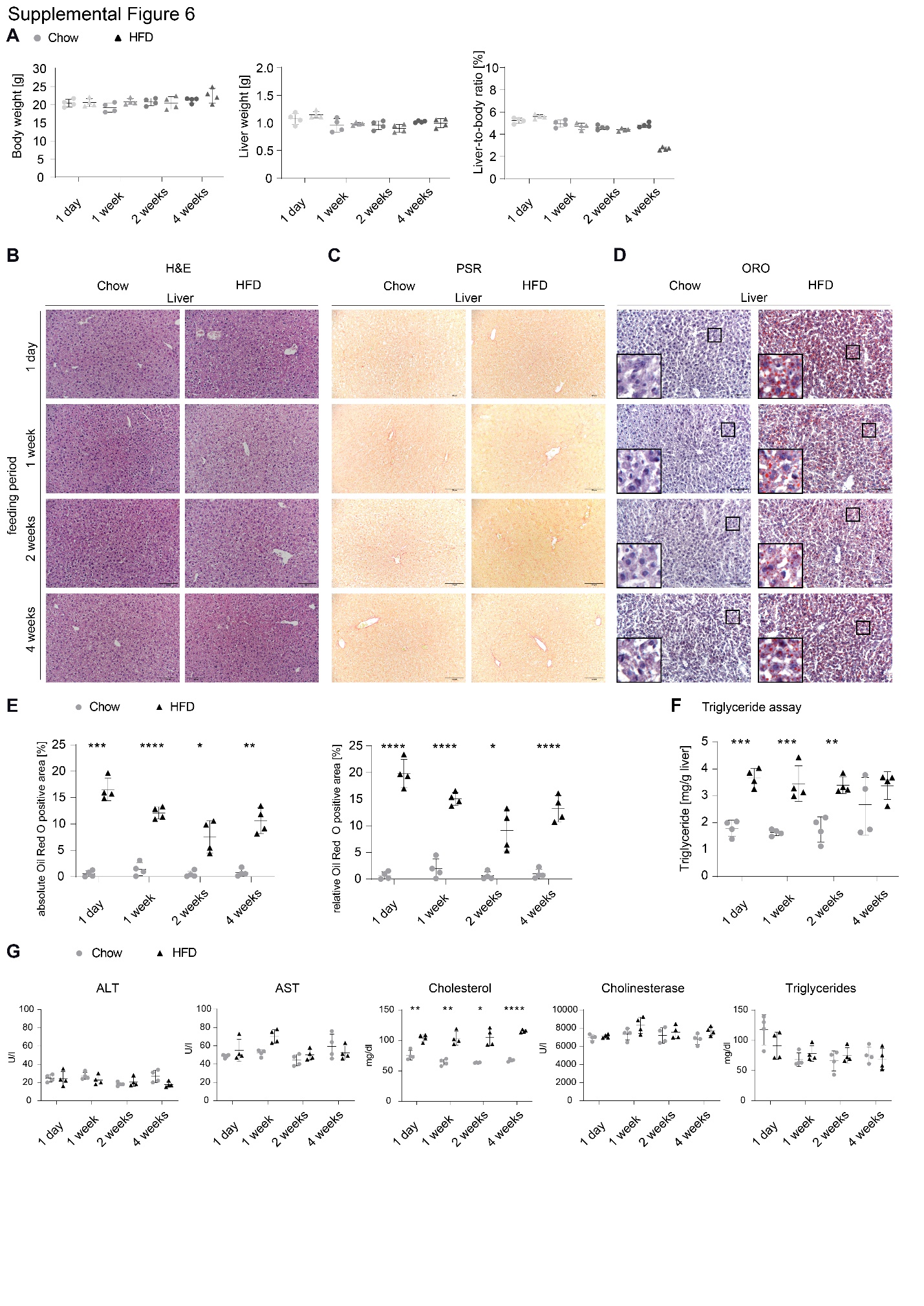
**

**Supplemental Figure 6. Hepatic HFD characterization at earlier feeding time points. (A)** Total body weights, liver weights and liver-to-body ratios during feeding periods of 1 day, 1, 2 and 4 weeks with n=4 per group (all groups, n.s., unpaired t-test). **(B-D)** Histopathological images of livers from chow and HFD-fed mice with **(B)** H&E staining (scale bars=100 µm), **(C)** PSR staining (scale bars=100 µm) and **(D)** ORO staining (scale bars=100 µm). ORO stainings are shown with enlarged sections to better visualise the intraparenchymal lipid accumulation. **(E)** Quantification of ORO positive percentage of absolute liver area (1 day, p=0.0003, unpaired t-test; 1 week, p<0.0001, unpaired t-test; 2 weeks, p=0.0173, unpaired t-test; 4 weeks, p=0.0025, unpaired t-test) and of relative liver area (1 day, p<0.0001, unpaired t-test; 1 week, p<0.0001, unpaired t-test; 2 weeks, p=0.0194, unpaired t-test; 4 weeks, p<0.0001, unpaired t-test). Relative quantified area was calculated by subtracting the white background and calculating the ratio of red staining (lipids) to blue staining (majority of hepatocytes). **(F)** Triglyceride assay of hepatic tissue (1 day, p=0.0002; 1 week, p=0.0003; 2 weeks, p=0.0011; 4 weeks, n.s., one-way ANOVA). **(G)** Plasma levels of ALT (all groups, n.s., unpaired t-test), AST (all groups, n.s., unpaired t-test), total cholesterol (1 day, p=0.0015, unpaired t-test; 1 week, p=0.0006, unpaired t-test; 2 weeks, p=0.0286, Mann-Whitney *U* test; 4 weeks, p<0.0001, unpaired t-test), cholinesterase (all groups, n.s., 1 day Mann-Whitney *U* test and 1, 2 and 4 weeks unpaired t-test) and triglycerides (all groups, n.s. 1 week Mann-Whitney *U* test and 1 day, 2 and 4 weeks unpaired t-test) in control mice and HFD-fed mice.

**
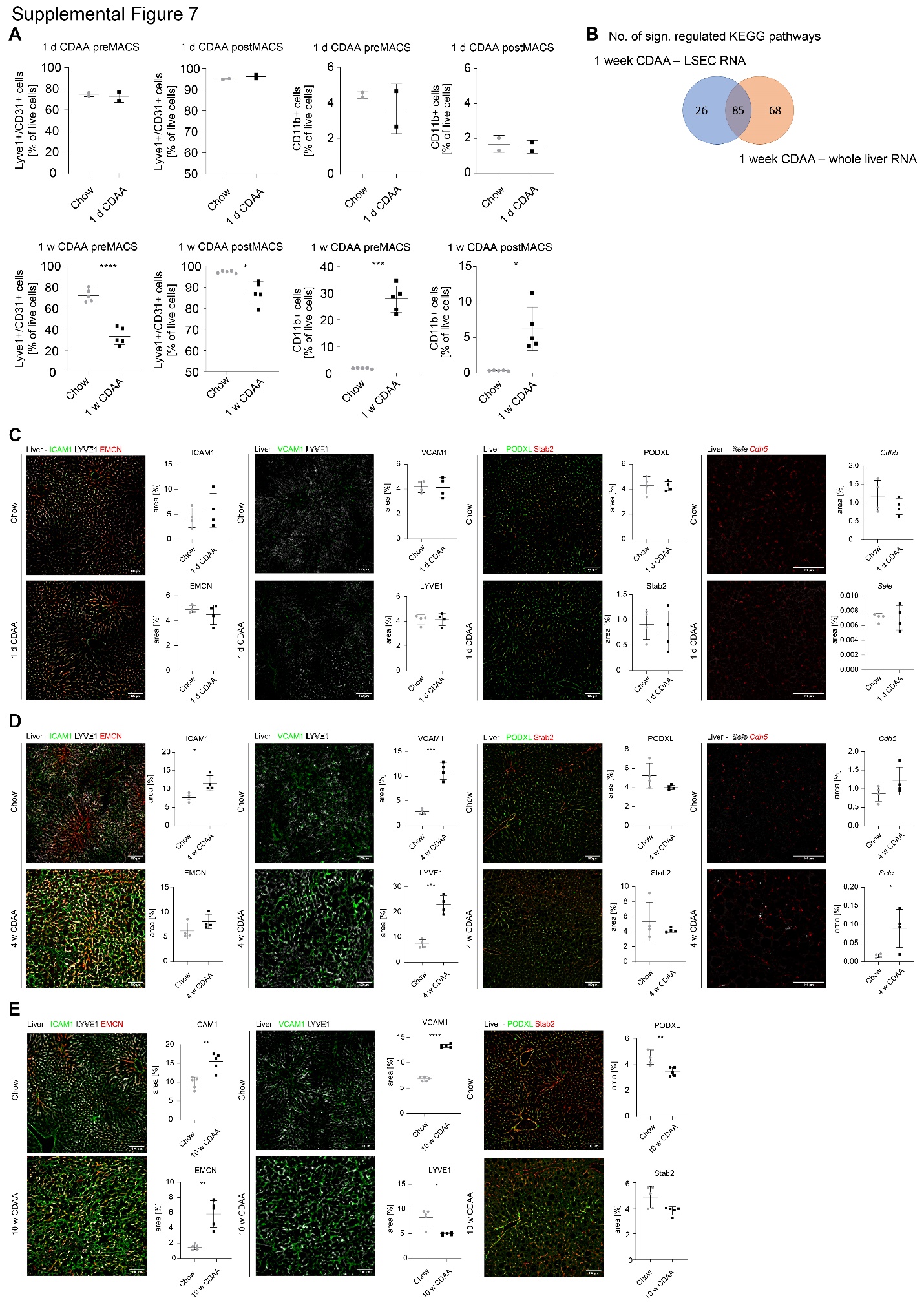
Supplemental Figure 7. Induction of macrophages during early phases of CDAA diet. (A)** Top 30 up-regulated

**Supplemental Figure 7. Purity of isolated LSEC and commonly dysregulated KEGG pathways in whole liver and isolated LSECs and endothelial cell adhesion molecules after 1 day, 4 and 10 weeks of CDAA diet. (A)** Lyve1+ and CD31+ as well as CD11b+ cells after 1 day and 1 week CDAA diet in preMACS and postMACS in percent of live cells (1d CDAA preMACS and postMACS, 2 vs. 2, n.s.; 1 w CDAA preMACS Lyve1+ and CD31+, 5 vs. 5, p<0.0001, unpaired t-test; 1 w CDAA postMACS Lyve1+ and CD31+, 5 vs. 5, p=0.0137, unpaired t-test; 1 w CDAA preMACS CD11b+, 5 vs. 5, p=0.0003, unpaired t-test; 1 w CDAA postMACS CD11b+, 5 vs. 5, p=0.0128, unpaired t-test). **(B)** Venn diagram showing common KEGG pathways in whole liver and isolated LSECs RNA Seq results, as well as non-common KEGG pathways of each group after 1 week of CDAA diet (Padj < 0.05). **(C-E)** IF staining, FISH and quantifications of adhesion molecules and LSEC marker in mouse livers after 1 day, 4 and 10 weeks of CDAA diet. **(C)** 1 day CDAA: ICAM1 (4 vs. 4, n.s., unpaired t-test), EMCN (4 vs. 4, n.s., unpaired t-test), VCAM1 (4 vs. 4, n.s., unpaired t-test), LYVE1 (4 vs. 4, n.s., unpaired t-test), PODXL (4 vs. 4, n.s., unpaired t-test) and Stab2 (4 vs. 4, n.s., unpaired t-test) as well as FISH of *Cdh5* (4 vs. 4, n.s., unpaired t-test) and *Sele* (4 vs. 4, n.s., unpaired t-test). **(D)** 4 weeks CDAA: ICAM1 (4 vs. 4, p=0,0164, unpaired t-test), EMCN (4 vs. 4, n.s., Mann-Whitney U test), VCAM1 (4 vs. 4, p=0,0001, unpaired t-test), LYVE1 (4 vs. 4, p=0,0002, unpaired t-test), PODXL (4 vs. 4, n.s., unpaired t-test) and Stab2 (4 vs. 4, n.s., unpaired t-test) as well as FISH of *Cdh5* (4 vs. 4, n.s., unpaired t-test) and *Sele* (4 vs. 4, p=0,0291, unpaired t-test). **(E)** 10 weeks CDAA: ICAM1 (4 vs. 4, p=0,0022, unpaired t-test), EMCN (4 vs. 4, p=0,0041, unpaired t-test), VCAM1 (4 vs. 4, p<0,0001, unpaired t-test), LYVE1 (4 vs. 4, p=0,0116, unpaired t-test), PODXL (4 vs. 4, p=0,0046, unpaired t-test) and Stab2 (4 vs. 4, n.s., Mann-Whitney *U* test).

**
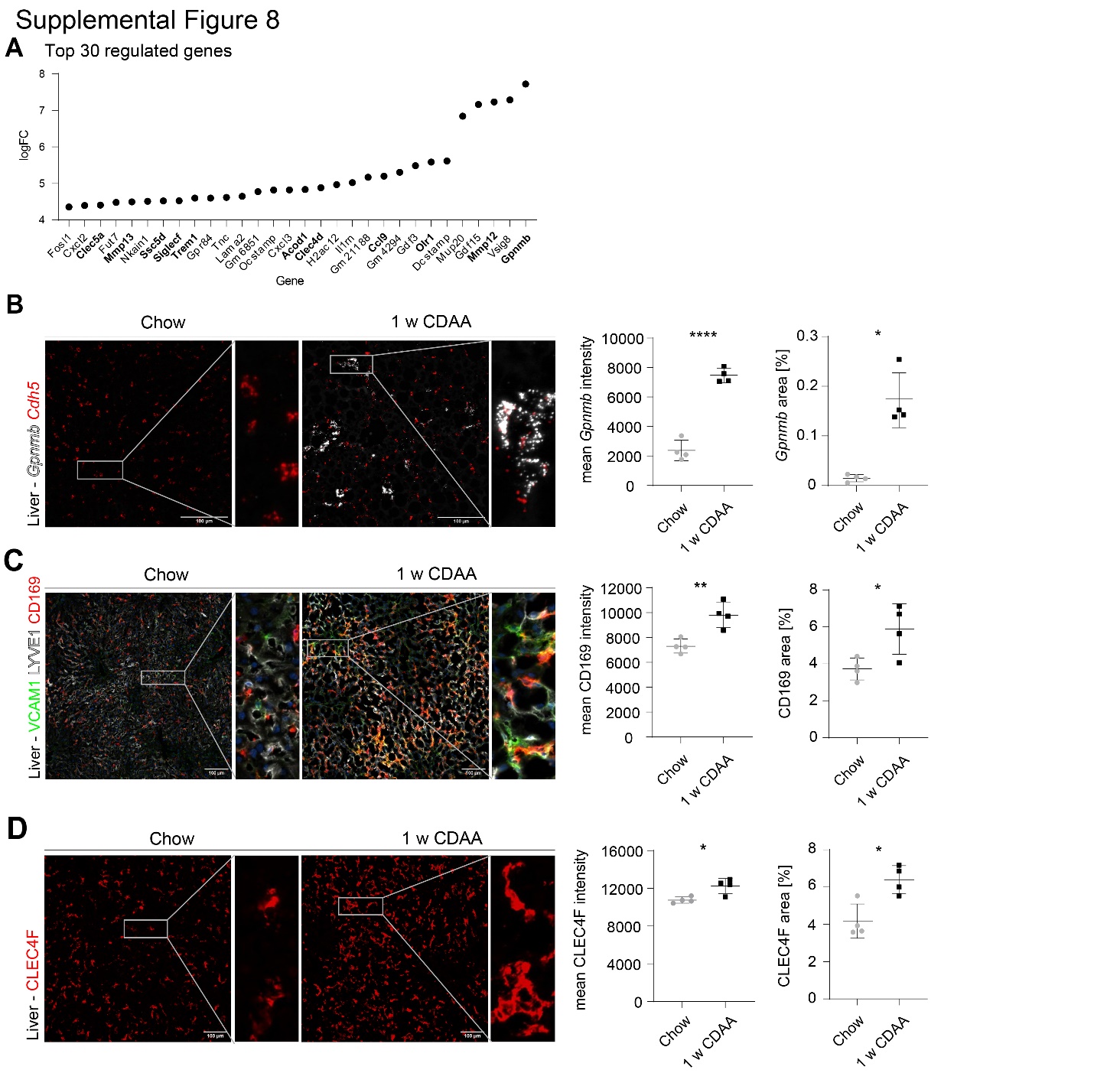
**

**Supplemental Figure 8. Induction of macrophages during early phases of CDAA diet. (A)** Top 30 up-regulated genes with macrophage genes shown in bold type in isolated LSECs after 1 week on a CDAA diet. **(B)** FISH of *Gpnmb* (4 vs. 4, mean intensity p<0.0001, unpaired t-test; mean area, p=0.01, unpaired t-test) and *Cdh5*. **(C-D)** IF staining (left side) and quantification (right side) of macrophage markers and adhesion molecules after 1 week of CDAA diet. **(C)** VCAM1, LYVE1 and CD169 (4 vs. 4, mean CD169 intensity, p=0.0092, unpaired t-test; mean CD169 area, p=0.0425, unpaired t-test). **(D)** CLEC4F (4 vs. 4, mean CLEC4F intensity, p=0.0258, unpaired t-test; mean CLEC4F area, p=0.0286, Mann-Whitney *U* test).

**
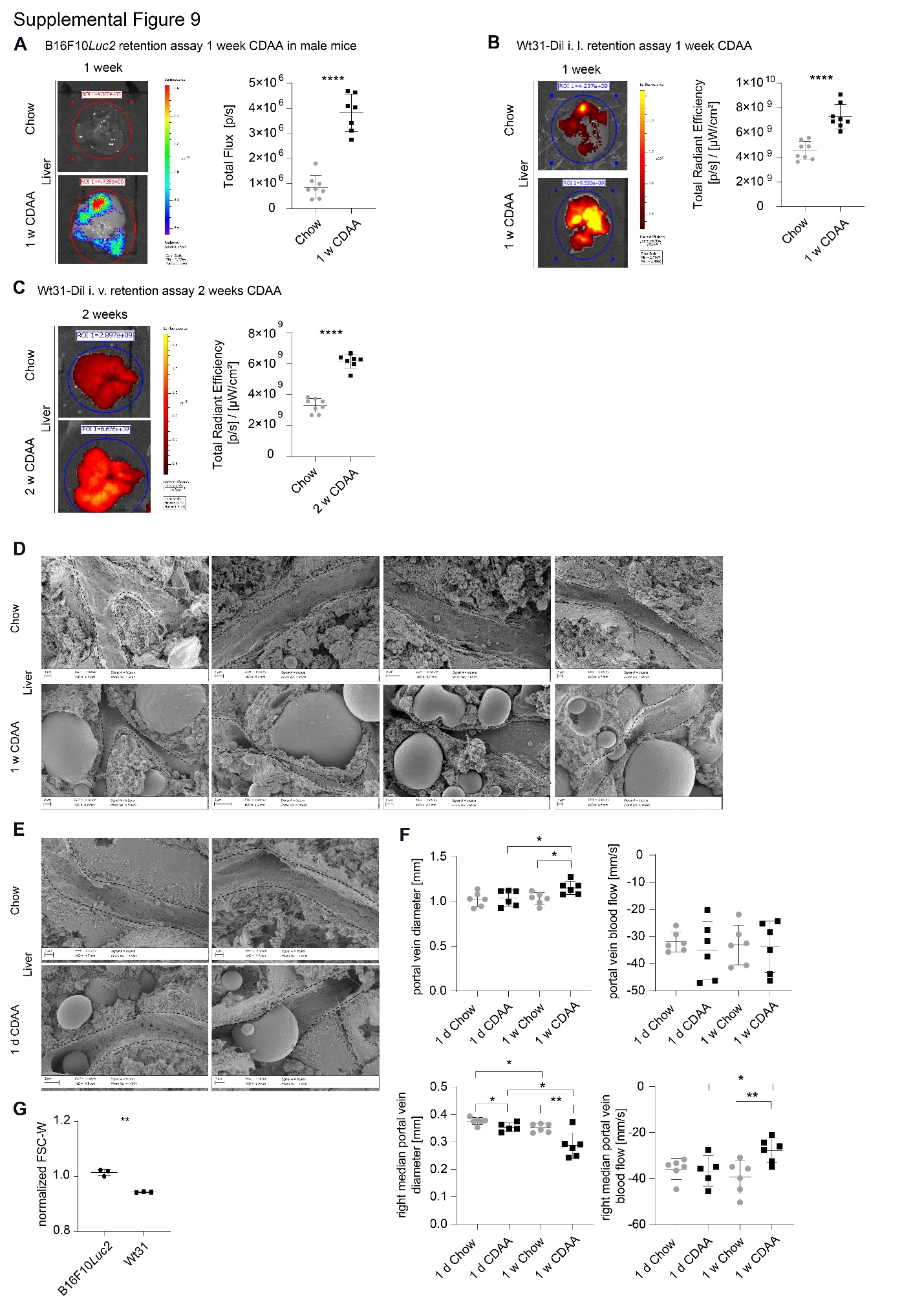
**

**Supplemental Figure 9. Additional retention assays using male mice or Dil-labeled Wt31 cells, scanning electron microscopy, liver echocardiography of mice fed the CDAA diet for 1 week and cell size comparison of B16F10*Luc2* and Wt31 melanoma cells. (A)** Cell retention assay of male mice fed the CDAA diet for one week. Left panel: *ex vivo* BLI images of livers 90 minutes after B16F10*Luc2* cell injection. Scale: Min: 5.57x10^4^ (p/sec/cm^2^/sr); Max: 1.63x10^5^ (p/sec/cm^2^/sr). Right panel: Livers were set as regions of interest and BLI was quantified. Quantification of BLI in livers (8 vs. 7, p<0.0001, unpaired t-test). **(B)** Left panel: *ex vivo* fluorescence images of livers 90 minutes after i.l. Wt31-Dil cell injection. Scale: Min: 2.79x 10^7^ ((p/sec/cm^2^/sr)/(µW/cm²)); Max:2.49 x 10^8^ ((p/sec/cm^2^/sr)/(µW/cm²)). Right panel: livers were set as regions of interest and fluorescence was quantified. Quantification of fluorescence in livers (8 vs. 8, p<0.0001, unpaired t-test). **(C)** Left panel: *ex vivo* fluorescence images of livers 90 minutes after i.v. Wt31-Dil cell injection. Scale: Min: 3.2x 10^7^ ((p/sec/cm^2^/sr)/(µW/cm²)); Max: 1.5 x 10^8^ ((p/sec/cm^2^/sr)/(µW/cm²)). Right panel: livers were set as regions of interest and fluorescence was quantified. Quantification of fluorescence in livers (8 vs. 7, p<0.0001, unpaired t-test). **(D, E)** Scanning electron micrographs of liver sinusoids from chow and 1 week (D) and from chow and 1 day (E) CDAA-fed mice ((D), n=5; one representative image selected per replicate, with two replicates shown Figure 8F, (E), n=3; one representative image selected per replicate, with one replicate shown Figure 8F). Dashed lines show the contour of the sinusoidal vessel walls in the chow and CDAA groups. **(F)** Echography-based assessment of external and internal liver vessel integrity. Portal vein analysis showing increased diameter (n=6 per group; 1 w Chow vs. 1 w CDAA p=0.0144, unpaired t-test; 1 d CDAA vs. 1 w CDAA p=0.0380, unpaired t-test) and preserved flow (n=6 per group, n.s., unpaired t-test), suggesting congestion of branched-off vessels. Right median portal veins (1^st^ generation branched-off vessels) show compressed diameter (n=6 per group, 1 d Chow vs. 1 d CDAA p=0.0328, unpaired t-test; 1 w Chow vs. 1 w CDAA p=0.0095, unpaired t-test; 1 d CDAA vs. 1 w CDAA p=0.0140, unpaired t-test; 1 d Chow vs. 1 w Chow p=0.0145, unpaired t-test) and reduced flow (n=6 per group, 1 w Chow vs. 1 w CDAA p=0.0088, unpaired t-test; 1 d CDAA vs. 1 w CDAA p=0.0318, unpaired t-test). **(G)** Flow cytometry quantification of normalised FSC-W for determination of relative cell size of B16F10*Luc2* and Wt31 (n=3, p=0.0084, unpaired t-test).
